# NIDS-β*: an explainable large language based framework for contextual intrusion resilience in network security

**DOI:** 10.3389/frai.2026.1746661

**Published:** 2026-03-16

**Authors:** Firas Saidi

**Affiliations:** 1 AI and Metaverse Research Centre (AIMRC), Department of Information Technology, College of Computer Science (CCS), University of Technology Bahrain, Salmabad, Bahrain; 2 RIADI Laboratory ENSI LR99ES26, University of Manouba, Tunisia

**Keywords:** LLM, network security, NIDS, NIDS-β*, SHAP analysis, XAI

## Abstract

Modern cyberattacks are escalating in scale and sophistication, driving the need for Network Intrusion Detection Systems (NIDS) that offer contextual reasoning, rapid adaptation, and operational transparency. In response to this challenge, this paper introduces NIDS-β*, a novel Large Language Model (LLM)-inspired framework that integrates deep context-aware analysis into the intrusion detection pipeline. Our approach synergizes transformer-based semantic embeddings with statistical flow features to jointly interpret network behavior quantitatively and contextually. Moreover, by incorporating Explainable AI (XAI) principles, NIDS-β* provides intrinsic interpretability through attention visualizations and SHapley Additive exPlanations (SHAP), yielding transparent and actionable alerts. Experimental results demonstrate that the proposed framework achieves strong performance, with a detection accuracy of 98.6 and 97.8%, on CIC-IDS2018 and UNSW-NB15 datasets, respectively. These results show that NIDS-β* consistently outperforms established Machine and Deep Learning baselines, including Decision Trees, CNN, BiLSTM, and Gradient Boosting Machines. Furthermore, experiments confirm robust zero-day attack resilience, attaining an F1-score of 0.972, alongside highly reliable model calibration reflected by an Expected Calibration Error of only 1.9% on CIC-IDS2018 dataset.

## 1 Introduction

The rapid growth of networked systems and cloud-based infrastructures has significantly expanded the attack surface of modern digital environments. As a result, cyberattacks are increasing not only in volume but also in sophistication, often exhibiting complex, multi-stage behaviors that evade traditional rule-based defenses (Goranin et al., 2025; Santos et al., 2025). Conventional signature-driven Network Intrusion Detection Systems (NIDS), while effective against known threats, struggle to generalize to previously unseen attack patterns and frequently suffer from high false-positive rates in dynamic traffic conditions (Neupane et al., 2022; Masdari and Khezri, 2020). These limitations motivate the development of intelligent detection mechanisms capable of contextual reasoning, adaptability, and reliable decision support.

Recent advances in Machine and Deep Learning (ML-DL) have improved intrusion detection by enabling automated feature extraction from network traffic. Models such as decision trees, ensemble learners, convolutional networks, and recurrent architectures have demonstrated promising results on benchmark datasets. Decision-tree and ensemble-based approaches effectively capture non-linear relationships in tabular flow features and offer relatively strong interpretability, making them attractive for early deployment scenarios. Convolutional and recurrent neural architectures further enhance detection performance by learning spatial and temporal patterns from traffic sequences, particularly in high-volume or structured attack scenarios (Hozouri et al., 2025; Trilles et al., 2024; Al-Haija et al., 2024). However, many of these approaches operate primarily on fixed, tabular representations and are limited in their ability to capture long-range temporal dependencies and higher-order relationships across heterogeneous network events. Moreover, the opacity of deep models often hampers their adoption in operational settings, where analysts require interpretable and trustworthy alerts.

In parallel, transformer-based architectures, originally developed for natural language processing, have demonstrated strong potential for NIDS by enabling contextual modeling of sequential network traffic. Although Large Language Models (LLMs) (Koubaa et al., 2023; Gupta et al., 2023) are often associated with generative tasks, their core self-attention mechanism is inherently well suited for learning contextual embeddings from ordered network events, such as flows, sessions, and protocol interactions. This insight has motivated recent research into LLM-inspired encoders for intrusion detection, where malicious behavior is frequently characterized by temporal dependencies and multi-stage patterns rather than isolated packets or individual features (Kasri et al., 2025; Saidi, 2025; Lian et al., 2025; Wali et al., 2025). However, many existing NIDS adaptations rely on generic language pre-training, introduce substantial computational overhead, or conflate generative and non-generative modeling paradigms, resulting in unclear system assumptions and limited operational suitability.

Motivated by these gaps, this paper introduces NIDS-β*, a transformer-inspired and explainable NIDS designed specifically for structured network traffic analysis. Rather than employing a generative or autoregressive language model, NIDS-β* adopts a compact, task-optimized Transformer encoder trained from scratch on network flow sequences. Network sessions are modeled as ordered token sequences derived from protocol attributes, statistical features, and discretized traffic descriptors, enabling the encoder to capture temporal dependencies and behavioral semantics across sessions. This contextual representation is combined with traditional tabular features to support accurate and robust intrusion detection.

To balance detection performance, robustness, and operational reliability, NIDS-β* employs a tiered multi-head detection architecture. Supervised classification heads handle known attack patterns, while a dedicated one-class anomaly detection head based on a Deep Support Vector Data Description (Deep-SVDD) objective enhances sensitivity to previously unseen or zero-day attacks. The outputs of these complementary detectors are fused and probability-calibrated to produce stable and trustworthy alerts. Importantly, the framework integrates eXplainable Artificial Intelligence (XAI) mechanisms, including attention-based token attribution and SHapley Additive exPlanations (SHAP), enabling analysts to understand both contextual and statistical factors underlying each detection decision.

The key contributions of this paper are summarized as follows:

- A task-specific contextual encoder for network traffic that leverages self-attention to model sequential dependencies without relying on autoregressive or text-generation capabilities.
- A tiered multi-head detection and fusion strategy is introduced that integrates supervised classifiers, ensemble-based models, and a one-class anomaly detection head to capture complementary aspects of network behavior, enabling robust detection of both known and zero-day attacks while maintaining low false-positive rates suitable for real-world deployment.
- Integrated explainability and operational reliability are achieved through intrinsic interpretability mechanisms, including attention-based visualization and SHAP-driven feature attribution, combined with calibrated probability estimates and alert de-duplication strategies that ensure stable and actionable intrusion detection in real-world operational settings.
- Comprehensive evaluation and benchmarking on the UNSW-NB15 and CIC-IDS2018 datasets with systematic comparison against representative ML, DL, and hybrid intrusion detection baselines, demonstrating strong detection accuracy, reliable probability calibration, low detection latency, and robust generalization to previously unseen attack categories.

The remainder of this paper is organized as follows. Section 2 surveys related work on machine learning, deep learning, and LLM-inspired approaches to network intrusion detection. Section 3 describes the architecture and algorithmic design of the proposed NIDS-β* framework in detail. Section 4 outlines the experimental setup and presents a comprehensive performance evaluation. Finally, Section 5 discusses the limitations of the proposed approach and concludes the paper by highlighting directions for future research.

## 2 Related work

Artificial intelligence–based techniques have become central to modern intrusion detection research, with ML and DL models forming the foundation of many state-of-the-art systems (Trilles et al., 2024; Al-Haija et al., 2024). These approaches leverage statistical and representation learning to automatically identify malicious patterns in network traffic. For instance, in Wong et al. (2025) authors propose an IDS based on Random Forest enhanced with XAI techniques to improve decision transparency and operational trust. Their approach integrates feature importance analysis and post-hoc explanations to justify classification outcomes while maintaining competitive detection accuracy. However, the framework relies on conventional ensemble learning and does not address temporal dependencies or adaptive learning under evolving attack behaviors. In Abdulganiyu et al. (2025), authors present a white-box intrusion detection model using decision trees combined with optimized feature selection to enhance interpretability and computational efficiency. The study demonstrates that carefully pruned trees can achieve strong detection performance while preserving full transparency of decision logic. Nonetheless, the model’s reliance on static tree structures limits its robustness against complex, high-dimensional, and evolving network traffic patterns.

In another recent work (Zhang et al., 2025), authors introduce XIDINTFL-VAE, a hybrid framework combining a variational autoencoder with XGBoost and class-wise focal loss to address severe class imbalance in intrusion detection. The method effectively enhances minority attack detection by leveraging latent representations and imbalance-aware optimization. Despite its strong performance, the architecture introduces increased complexity and lacks intrinsic explainability at the latent representation level.

In Shoukat et al. (2025), authors develop a dual-attention CNN-BiLSTM model that jointly captures spatial feature correlations and temporal traffic dynamics for network intrusion detection. The attention mechanisms improve feature discrimination and sequence modeling, leading to high detection accuracy across multiple attack categories. However, the deep architecture operates largely as a black box and requires substantial computational resources, which may hinder real-time deployment.

Recently, authors in Bizzarri et al. (2022), propose an explainable DL–based intrusion detection system tailored for industrial network environments, aiming to enhance trust and transparency in threat detection. Their framework integrates XAI mechanisms to interpret deep model decisions while achieving high detection accuracy on industrial traffic scenarios. Nevertheless, the approach remains largely dependent on post-hoc explainability and does not explicitly address probabilistic calibration, alert reliability, or generalization under continuously evolving attack patterns.

Others works, have explored diverse paradigms ranging from neuro-symbolic reasoning and representation learning to online anomaly detection and dataset-driven evaluation. Neuro-symbolic NIDS frameworks proposed by Bizzarri et al. (2022) and Wu et al. (2025) integrate Deep Neural models with symbolic logic to improve interpretability and open-set recognition, enabling systems to reason about unseen attacks through explicit rules and logical constraints. While effective in enhancing explainability and handling unknown classes, such approaches often require handcrafted knowledge bases or predefined logical structures, which may limit scalability and adaptability in highly dynamic network environments. In parallel, transformer-based representation learning has gained prominence, exemplified by ET-BERT (Mirsky et al., 2018), which is based on pre-trained transformers to model contextualized datagram representations for encrypted traffic classification; however, these models primarily focus on representation quality and classification accuracy, offering limited analyst-facing interpretability. Earlier anomaly-detection systems such as Kitsune (Sharafaldin et al., 2018) employ ensembles of autoencoders to achieve lightweight, online detection with low latency, but provide coarse anomaly scores without contextual or semantic explanations. Complementing these modeling efforts, the UNSW-NB15 dataset introduced by Qi et al. (2023) has become a foundational benchmark by characterizing modern attack behaviors and enabling systematic evaluation of ML- and DL-based NIDS. Collectively, these works highlight the trade-offs between interpretability, contextual reasoning, adaptability, and operational feasibility, motivating the need for frameworks that combine contextual sequence modeling, robust anomaly detection, and actionable explainability.

Moreover, LLMs have demonstrated significant potential across a wide range of application domains, with growing interest in their adaptation to cybersecurity and intrusion detection tasks. In Marques et al. (2023) introduced LogGPT, a log-based anomaly detection framework built on ChatGPT. This model explores the transferability of knowledge from large-scale corpora to log-based anomaly detection by utilizing ChatGPT’s language interpretation capabilities. Evaluated on the BGL and Spirit datasets, LogGPT’s performance was compared against three deep learning-based techniques. Similarly, in Petrović (2023), researchers investigated ChatGPT’s natural language capabilities to simplify the configuration of network simulation scenarios, demonstrating its utility for beginners in networking security by outlining procedures for setting up development and network analysis environments. Another study in Ali and Kostakos (2023), proposed a dialogue-like method using ChatGPT, where labeled data serves as context, and log records are assessed through questions to detect suspicious activity. While this approach shows promise for real-time DevSecOps scenarios, it faces limitations such as restricted context, processing time, cost, and lower accuracy. In Ullah et al. (2024), developed HuntGPT, a specialized intrusion detection dashboard integrating a Random Forest classifier with the KDD’99 dataset and explainable AI (XAI) frameworks such as SHAP and LIME have been combined with GPT-3.5 Turbo to present detected threats in an understandable and human-centric format. This framework demonstrates how conversational agents powered by LLMs and XAI techniques can deliver reliable, interpretable, and practical solutions for intrusion detection, thereby enhancing user comprehension and interaction.

In Maasaoui et al. (2024) proposed a contrastive learning combined with Bayesian Gaussian Mixture Model (BGMM), to learn discriminative representations that better separate normal and malicious traffic, particularly for rare attack classes. Experimental evaluations on modern benchmarks including UNSW-NB15 and CIC-IDS2017 demonstrate that such approaches can achieve competitive accuracy and F1-scores, highlighting their effectiveness in addressing feature representation and imbalance challenges in contemporary NIDS.

Another study Entezami et al. (2025), proposed a hybrid anomaly-detection framework that fine-tunes a pre-trained LLM for contextual analysis of security logs through tokenization and embeddings, alongside CNN/LSTM models applied to structured traffic features. The framework was evaluated on the KDD’99, UNSW-NB15, and CICIDS datasets collected from IDS and firewall sources. Experimental results indicate that the hybrid approach achieves approximately ~95% accuracy, precision of 0.92, recall of 0.90, an AUC of 0.95, an average detection latency of ~45 ms, and a false-alarm rate of 5%, outperforming both LLM-only and deep-learning-only baselines.

In Adjewa et al. (2025), proposed a hybrid anomaly-detection framework that integrates LLMs with DL techniques for enhanced network security monitoring. The framework exploits the contextual reasoning capability of LLMs alongside deep neural models to capture both semantic and traffic-level patterns in network data. Experimental evaluation reported detection accuracy around 95%, with improvements in precision and recall compared to standalone deep-learning baselines. The results demonstrate that LLM-assisted architectures can effectively strengthen anomaly detection performance while improving robustness against complex and evolving network threats. Similarly, García et al. (2025), propose an LLM-based continuous intrusion detection framework designed for next-generation network environments. Their approach encode network events into contextual representations, enabling adaptive detection across evolving traffic patterns. However, the framework primarily focuses on representation learning and continuous monitoring, with limited discussion on explainability, calibration reliability, and deployment-level efficiency under real-time constraints.

A recent work, Li et al. (2025) has begun to evaluate tabular foundation models as plug-and-play predictors for intrusion detection, emphasizing strong generalization with minimal task-specific training. Authors systematically benchmark TabPFN and TabICL on tabular intrusion detection settings and highlight both the promise and limitations of foundation-style priors under realistic IDS conditions.

Another work (Li et al., 2025), proposes an LLM-assisted IDS design that combines retrieval augmented generation with feature engineering and decision logic to improve adaptability and analyst interaction. These directions are complementary to NIDS-β*: while NIDS-β* learns a compact task-specific contextual encoder for flow sequences and couples it with calibrated multi-head detection, tabular foundation models and RAG-based IDS pipelines offer alternative paths to rapid adaptation and knowledge injection.

Despite the promising applications of LLMs in intrusion detection, several technical and practical limitations persist in existing research. These challenges underscore the need for further refinement to enable broader adoption and effectiveness in cybersecurity. First, the limited context window of ChatGPT constrains the volume of data that can be processed in a single inference, posing difficulties in scenarios requiring extensive historical context or large-scale traffic analysis. Second, although these models exhibit strong reasoning capabilities, their intrusion-detection accuracy remains suboptimal in some settings, with false positives and false negatives posing significant risks in high-stakes environments. Additionally, approaches such as the dialogue-based method rely heavily on labeled data for both training and contextual prompting, which is difficult to obtain due to the dynamic and evolving nature of cyber threats. This reliance restricts generalization to unseen attack patterns, highlighting critical gaps that must be addressed to advance LLM-based solutions for network security.

## 3 NIDS-β* framework for intrusion detection

This section describes the design and operation of the proposed NIDS-β* framework for intelligent intrusion detection. The framework adapts LLM-inspired principles contextual reasoning, sequential modeling, and deep representation learning to network traffic analysis by modeling flows as sequences of tokenized events, enabling the capture of temporal dependencies and behavioral semantics across sessions. In addition to accurate real-time detection, NIDS-β* integrates a dedicated control and adaptation plane for performance monitoring, calibration, and drift handling, while employing a compact, task-optimized Transformer encoder to ensure explainability, stability, and low-latency deployment in dynamic network environments.

### 3.1 End-to-end detection pipeline

**Figure 1:**
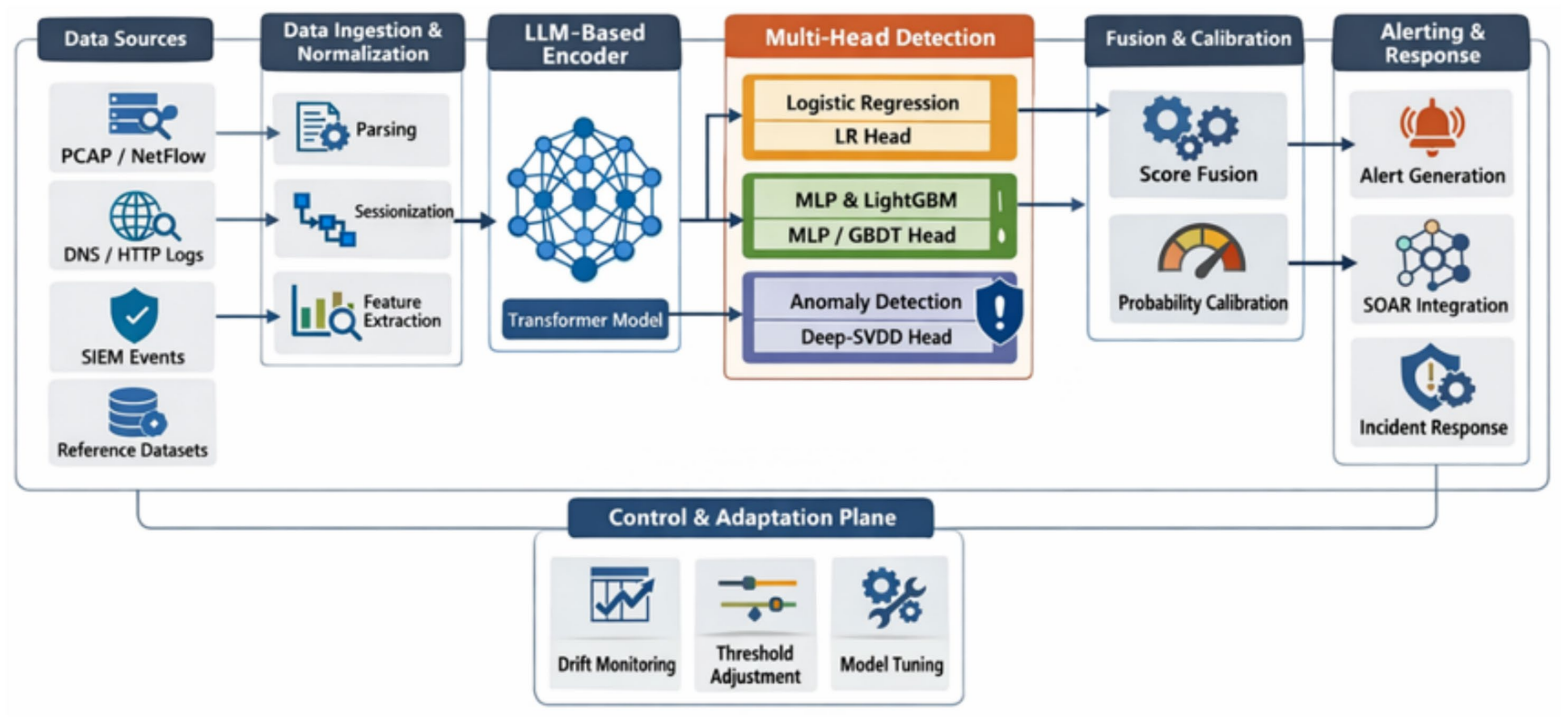
End-to-end pipeline of NIDS-β*.

The proposed NIDS-β* framework follows the end-to-end pipeline illustrated in Figure 1, consisting of the following sequential stages:

The pipeline ingests heterogeneous network telemetry, including PCAP/NetFlow records, DNS and HTTP(S) logs, firewall and proxy logs, SIEM events, and reference intrusion datasets. In this work, the UNSW-NB15 and CIC-IDS2018 datasets serve as the primary data source, providing labeled benign and malicious traffic representative of real-world attack scenarios.

#### 3.1.1 Stage 1. Data ingestion and normalization

Raw traffic records are parsed and normalized through de-duplication, timestamp synchronization, schema alignment, and Personally Identifiable Information (PII) scrubbing. This stage produces canonical flow-level events with consistent fields such as 5-tuple identifiers, protocol flags, payload sizes, and optional higher-layer attributes (e.g., HTTP headers or TLS/JA3 fingerprints). Normalized events are grouped into logical sessions using direction-agnostic 5-tuple identifiers and inactivity thresholds. Sliding windows of 30–120 s are applied to derive tabular features, including packet and byte counts, inter-arrival statistics, round-trip times, entropy measures, and application-level descriptors.

#### 3.1.2 Stage 2. LLM-based contextual encoding

In parallel with tabular feature extraction, session events are converted into ordered token sequences, where categorical attributes are mapped to discrete tokens and variable-length fields (e.g., domains and URIs) are encoded using subword tokenization. These sequences are processed by a compact Transformer-based encoder, trained from scratch on network traffic data, to generate dense contextual embeddings capturing temporal and behavioral dependencies.

#### 3.1.3 Stage 3. Multi-head detection

The learned embeddings and tabular features are evaluated by multiple detection heads operating in parallel:

- The Logistic Regression (LR) head, positioned as the always-on detection path in the pipeline, operates solely on lightweight tabular features (e.g., ct_* statistics, byte counts, durations, and protocol flags), ensuring continuous, low-latency protection and serving as a reliable fallback when the Transformer encoder is unavailable due to rate limiting or partial telemetry loss.
- Multi-Layer Perceptron (MLP) head activation occurs when contextual embeddings are produced by the encoder, enabling the fusion of Transformer-derived embeddings with engineered tabular features to capture non-linear and context-dependent attack patterns that cannot be represented by linear decision boundaries. Downstream in the pipeline, the outputs of the LR and MLP heads are probability-calibrated and fused, ensuring that detection scores remain stable and comparable under evolving traffic conditions.
- LightGBM head integration further strengthens the pipeline’s resilience by providing a high-capacity tabular learner capable of modeling heterogeneous and imbalanced traffic, while a Deep-SVDD–based one-class head operates in parallel on Transformer embeddings to maintain continuous sensitivity to anomalous and previously unseen behaviors.

#### 3.1.4 Stage 4. Fusion and calibration

Outputs from all detection heads are normalized and combined using a weighted linear fusion strategy. The fused score is subsequently calibrated to produce stable and reliable intrusion probabilities across varying traffic conditions.

#### 3.1.5 Stage 5. Control and adaptation plane

Operating alongside the detection pipeline, the Control and Adaptation Plane continuously monitors model behavior through drift detection, threshold adjustment, and periodic model tuning. This plane ensures long-term robustness, explainability, and operational stability under evolving network conditions.

#### 3.1.6 Stage 6. Alerting and response

Calibrated detection scores are post-processed through smoothing, de-duplication, and correlation mechanisms before triggering alerts. Generated alerts are enriched with explanations and are designed to integrate seamlessly with downstream SOAR and incident response systems.

### 3.2 Deep-SVDD on transformer embeddings

As shown in the multi-head detection stage of the pipeline (Stage 3 in Figure 1), NIDS-β* complements supervised detection with a continuously active anomaly detection path to address previously unseen and zero-day attacks. While the supervised heads focus on classifying known attack patterns, the one-class detection head operates independently of labeled attack data and provides an orthogonal detection signal based on deviations from learned normal behavior. The following subsection details the design and training of this Deep-SVDD–based one-class detection mechanism, which is tightly integrated with the Transformer embedding space produced by the contextual encoder.

Rather than employing a kernel-based One-Class Support Vector Machine (OC-SVM), NIDS-β* adopts a Deep-SVDD–style one-class objective operating on the learned Transformer embeddings. This approach learns a compact representation of benign traffic by minimizing the distance between embeddings of normal samples and a reference center in latent space. During inference, flows or sessions whose embeddings deviate significantly from this center are assigned high anomaly scores and flagged as potentially malicious.

This Deep-SVDD formulation preserves the core principle of one-class learning while avoiding the scalability and kernel-selection limitations of classical OC-SVMs in high-dimensional embedding spaces (see Table 1). It is particularly suitable for NIDS scenarios, where attack data are sparse, diverse, and continuously evolving, and where modeling the statistical profile of normal traffic (e.g., packet counts, flow durations, protocol flags, and byte distributions) is critical for robust zero-day detection.

**Table 1:** Comparison of OCM-SVM and Deep-SVDD–style for NIDS.

Aspect	OC-SVM-based NIDS	Deep-SVDD-style in NIDS-β*
Core principle	Kernel-based boundary enclosing normal traffic	Neural objective learning compact normal embedding
Feature space	Fixed, hand-engineered features	Learned high-dimensional embeddings
Representation learning	Not supported	Jointly learned with encoder
Scalability to high dimensions	Limited (kernel sensitivity)	Designed for high-dimensional latent spaces
Compatibility with Transformer/LLM encoders	Indirect and inefficient	Native and seamless
Kernel/hyperparameter sensitivity	High (kernel, γ, ν)	Low (distance-based objective)
Training complexity	O(n^2^–n^3^) in worst case	O(n) per epoch
Zero-day/unseen attack detection	Moderate	Strong
Robustness under evolving traffic	Degrades with drift	Adaptable via representation updates
Integration with multi-head fusion	Awkward	Clean and modular
Interpretability	Moderate (support vectors)	Moderate (distance + embedding analysis)
Suitability for real-time deployment	Limited at scale	Well-suited
Typical role in modern NIDS	Baseline/legacy reference	Primary anomaly head
Use in NIDS-β*	Conceptual baseline only	Operational one-class head

### 3.3 Training objective of the one-class head

The one-class detection head is trained using a Deep-SVDD–style objective, where only benign samples are used during optimization. Let z(x)denote the embedding produced by the Transformer encoder for input x. The objective minimizes the squared distance between z(x)and a reference center c in latent space (Equation 1):


$${ℒ}_{\text{SVDD}}=∥z(x)-c{∥}_{2}^{2}$$ (1)

During inference, samples whose distance exceeds a learned threshold are assigned high anomaly scores and contribute to zero-day intrusion detection. This formulation enables seamless integration with the learned representation space and avoids the scalability limitations of kernel-based OC-SVM in high-dimensional settings.

While the supervised and one-class detection heads in NIDS-β* provide complementary views of network behavior, their outputs must be combined in a principled manner to produce stable and actionable intrusion decisions. Direct reliance on any single head may lead to brittle behavior under distribution shift or incomplete context, particularly in dynamic network environments. To address this, NIDS-β* employs a dedicated fusion and optimization strategy, described next, which integrates calibrated outputs from all detection heads into a unified and reliable decision score.

### 3.4 Fusion strategy and optimization

The outputs of the detection heads are combined through a weighted linear fusion scheme. Each head is trained independently using its respective objective: cross-entropy loss for the LR and MLP heads, gradient-boosting loss for LightGBM, and a Deep-SVDD–style one-class objective for the anomaly detection head. After training, the outputs of all heads are normalized and probability-calibrated using Platt scaling or isotonic regression on a held-out validation set.

Fusion weights are then learned offline by minimizing the negative log-likelihood of the fused prediction on validation data, subject to non-negativity and simplex constraints to ensure interpretability and stability. This process yields a fixed set of fusion weights used during inference. As a result, the detection heads are not optimized jointly end-to-end, which simplifies training, improves modularity, and allows individual heads to be updated or replaced without retraining the entire system.

### 3.5 Compact fusion formulation

Let $p_{i}(x)$denote the calibrated output of detection head i. The final detection score is computed as in Equation 2 below:

$$\hat{p}(x)=\sum_{i=1}^{K}w_{i}\;p_{i}(x),\sum_{i}w_{i}=1,w_{i}\geq 0$$ (2)

where the fusion weights $w_{i}\;$are optimized on validation data.

While the detection pipeline and fusion strategy described above produce calibrated and reliable intrusion decisions at inference time, operational deployment requires continuous oversight to maintain performance, trust, and stability under evolving network conditions. Intrusion detection systems must not only generate accurate predictions but also provide transparent explanations, adapt to concept drift, and incorporate analyst feedback over time. To address these requirements, NIDS-β* introduces a dedicated Control and Adaptation Plane, described in the following subsection, which supervises explainability, drift monitoring, active learning, and continual optimization alongside the core detection pipeline.

### 3.6 Control and adaptation plane

**Figure 2:**
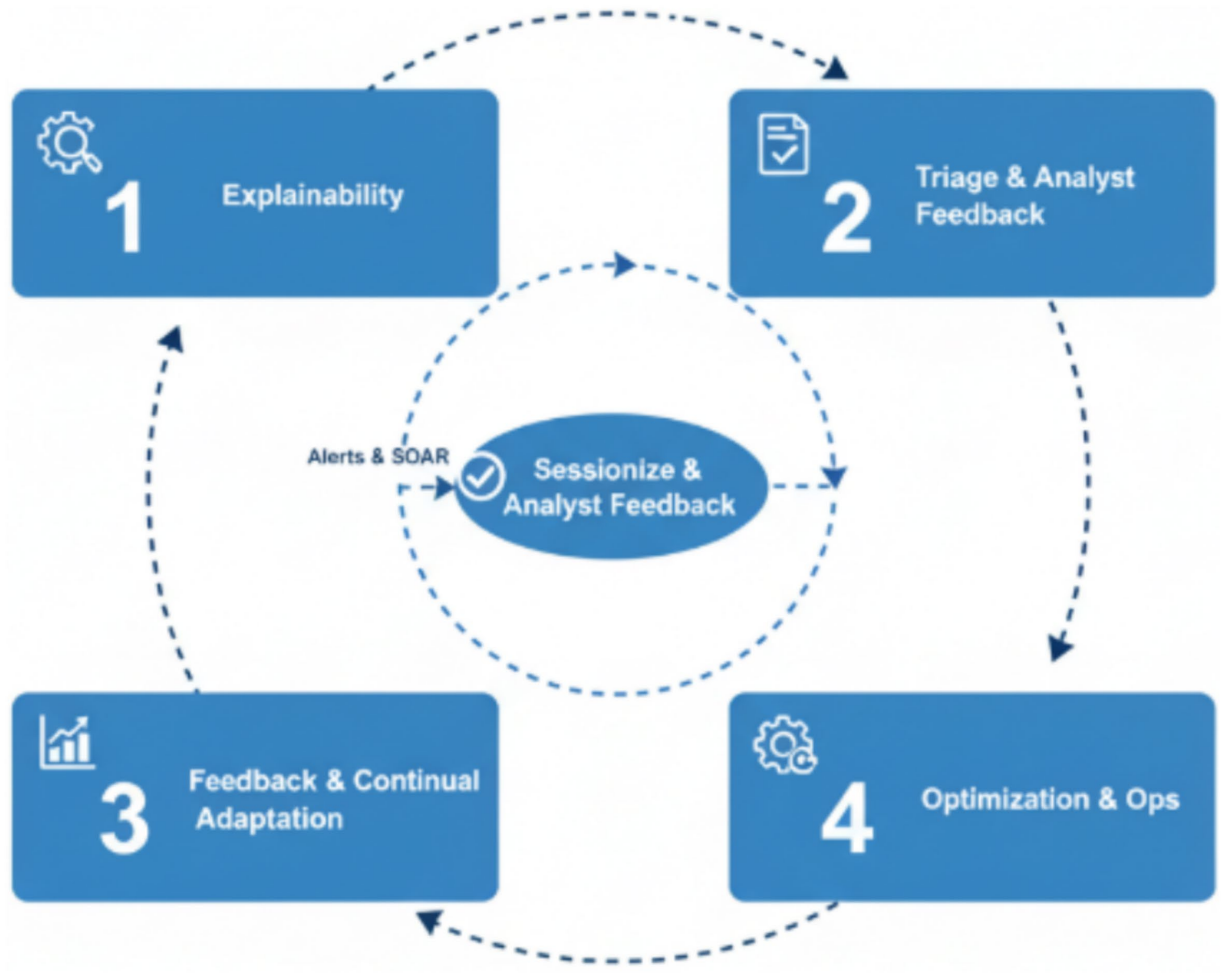
Control and adaptation plane pipeline of NIDS-β*.

The Control and Adaptation Plane (Figure 2) is presented as an architectural component designed to support long-term deployment through drift monitoring, analyst feedback capture, and periodic model maintenance. In the current study, we focus experimental validation on the core detection pipeline (encoding, multi-head detection, fusion/calibration, and explainability generation). The adaptation-plane mechanisms (e.g., drift detectors, active learning loops, continual tuning guardrails) are therefore described as design elements and are not separately benchmarked in this paper; we discuss their evaluation as part of future deployment-oriented work.

Triage and analyst feedback employ a risk score tuned on UNSW-NB15 and CIC-IDS2018 validation curves, with suppression logic for burst control and “known-good” patterns; analyst dispositions (TP/FP/U) are captured to provide learning signals.

For feedback and continual adaptation, the framework employs active learning through margin sampling to identify and label the most uncertain UNSW-NB15 and CIC-IDS2018 -like samples, ensuring the model remains sensitive to evolving traffic behaviors. Concept drift is monitored via two complementary mechanisms:

1. Kullback–Leibler (KL) divergence: an information-theoretic measure quantifying how one probability distribution diverges from another, computed here over token and feature distributions to capture subtle shifts in data semantics and statistical characteristics;
2. ADWIN (ADaptive WINdowing): an online, parameter-free drift detector that dynamically adjusts the size of its sliding window and signals drift when recent statistics differ significantly from historical observations beyond a confidence threshold. Together, KL divergence provides fine-grained distributional sensitivity, while ADWIN offers robust, real-time detection of abrupt or gradual drifts.

Detection heads are periodically re-fitted, and when deployed on-premise, mini fine-tuning sessions of the distilled encoder are scheduled with strict guardrails such as freezing lower layers and incorporating replay mixing with baseline UNSW-NB15 samples to mitigate catastrophic forgetting. The Optimization and Ops layer ensures dependable deployment through LLM-to-small-encoder distillation, quantization, request batching, and caching strategies, balancing inference efficiency with continual model reliability.

### 3.7 NIDS-β* algorithmic design

The Algorithm 1 orchestrates all the components aforementioned and discussed in subsection 3.1 within a unified end-to-end pipeline, enabling real-time, low-latency streaming inference. This cohesive architecture seamlessly integrates data ingestion, feature engineering, contextual encoding, and multi-head detection culminating in a system that transforms raw telemetry into interpretable, calibrated, and actionable intrusion alerts.

ALGORITHM 1: NIDS-β* Algo

**Figure:**
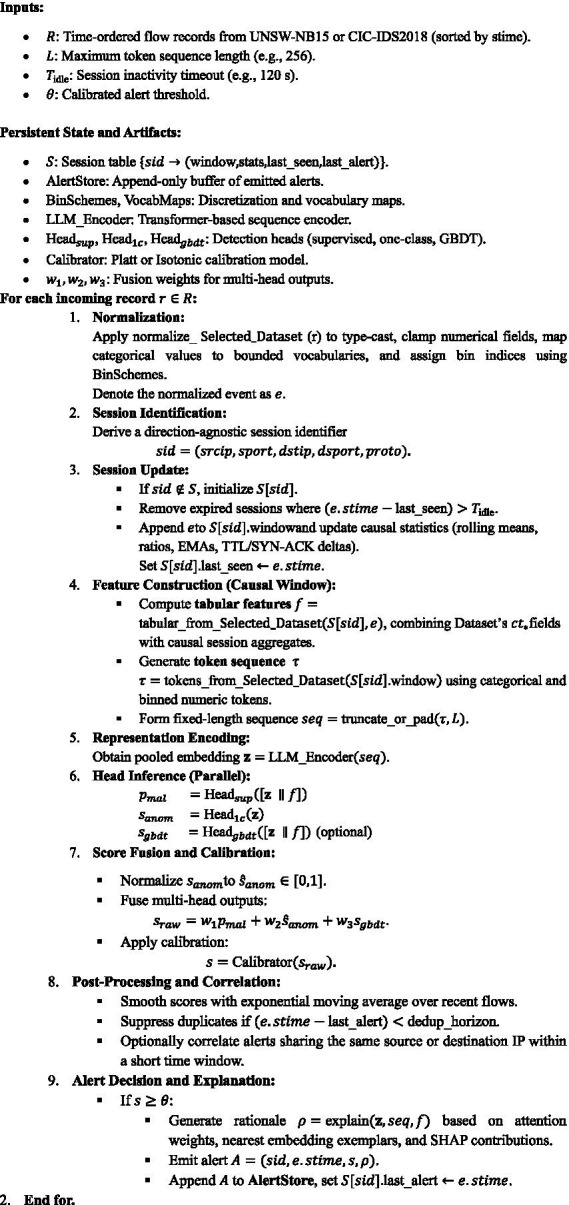

The algorithm 1 operates in a fully causal streaming mode, where each network flow record is processed sequentially as it would arrive in a live network environment. It begins with receiving a time-ordered stream of flow records R, each containing features such as source and destination IP addresses, ports, protocol, and a set of pre-engineered statistical indicators (the *ct_* fields). For every incoming record r, a normalization step is applied through the function normalize_ “Selected_Dataset” (r). This operation type-casts all numerical fields, clamps outliers to avoid heavy-tailed distortions, maps categorical values (e.g., protocols, services, states) to bounded vocabularies defined by *VocabMaps*, and discretizes continuous variables into bins according to *BinSchemes*.

The resulting standardized event is denoted e. Next, the algorithm establishes a direction-agnostic session identifier sid=(srcip,sport,dstip,dsport,proto)that links flows belonging to the same logical communication pair. The session table Smaintains an entry for each active session, consisting of a rolling window of recent flows, incremental statistics (e.g., mean byte ratios, inter-arrival times, SYN/ACK dynamics), and timestamps of the last observed and last alerted events. If the identified session does not exist, it is initialized; otherwise, its record is updated. Any session whose inactivity exceeds the configured timeout $T_{\text{idle}}$is purged to maintain bounded memory usage.

From the updated causal session window, two complementary representations are derived.

- First, a tabular feature vector f=tabular_from_UNSW(S[sid],e)is computed, combining UNSW or CIC-IDS’s engineered *ct_* statistics with causal, session-level aggregates such as byte/packet exponential moving averages (EMAs), TTL deviations, and directional traffic ratios.
- Second, a token sequence τ=tokens_from_UNSW(S[sid].window)is generated, where categorical attributes are converted into symbolic tokens and numerical attributes are represented by their bin identifiers. Event markers (e.g., *SYN_only*, *HTTP_method_seen*) are also included to capture semantic context. The resulting sequence is truncated or padded to a fixed length L, forming seq.

This hybrid representation is then encoded by a Transformer-based large language model encoder, z=LLM_Encoder(seq), which produces a pooled embedding capturing both short-term dependencies and higher-order semantic patterns in the network flow. The embedding zand tabular features fare subsequently processed by three specialized inference heads operating in parallel:

1. Supervised Head (${\text{Head}}_{\sup}$): computes $p_{\mathrm{mal}}={\text{Head}}_{\sup}([z∥f])$, representing the probability of a known intrusion pattern based on labeled data.
2. One-Class Head (${\text{Head}}_{1c}$): computes $s_{\text{anom}}={\text{Head}}_{1c}(z)$, providing an anomaly score trained exclusively on normal traffic to enhance sensitivity to zero-day threats.
3. GBDT Head (${\text{Head}}_{\text{gbdt}}$): optionally evaluates $s_{\text{gbdt}}={\text{Head}}_{\text{gbdt}}([z∥f])$, exploiting gradient-boosted decision trees for robust modeling of complex tabular relationships.

The outputs of the three heads (Equation 3) are linearly fused using learned weights $w_{1},w_{2},w_{3}$:

$$s_{\mathrm{raw}}=w_{1}p_{\mathrm{mal}}+w_{2}{\hat{s}}_{\text{anom}}+w_{3}s_{\text{gbdt}}$$ (3)

where ${\hat{s}}_{\text{anom}}$is the normalized anomaly score. The fused score is then calibrated through a pre-trained mapping (Platt or isotonic regression) to produce a well-calibrated probability $s=\text{Calibrator}(s_{\mathrm{raw}})$ within [0,1].

A post-processing stage follows to ensure operational stability. The calibrated scores are smoothed over time using an exponential moving average to mitigate transient spikes. Duplicate alerts within a defined temporal horizon are suppressed, and related alerts (e.g., sharing the same source or destination IP) are correlated to form higher-level incidents.

Finally, when the smoothed score exceeds the alert threshold s≥θ, the algorithm triggers the alert generation and explanation stage. Here, the function explain (𝑧, seq, f) produces a human-readable rationale ρby integrating three interpretability layers:

- Layer 1: Attention heat-maps from the LLM encoder indicating which tokens influenced the prediction most strongly,
- Layer 2: Exemplar retrieval highlighting similarity to known attack or benign patterns in the embedding space, and
- Layer 3: SHAP-based feature attributions revealing the tabular factors contributing most to the decision.

The final alert object A is appended to the AlertStore, and the session’s last alert timestamp is updated. Through this sequence of operations, Algorithm 1 enables adaptive, explainable, and real-time network intrusion detection. It integrates sequential reasoning inspired by large language models with tabular feature learning and anomaly detection mechanisms, achieving a balanced trade-off between accuracy, interpretability, and zero-day detection capability.

## 4 Experiments and evaluation

Experiments are conducted using publicly available benchmark datasets, mainly UNSW-NB15 and CIC-IDS2018 datasets. This dataset provides a modern, realistic representation of network traffic labeled with contemporary attack categories.

### 4.1 Dataset and pre-processing

The experimental validation of the proposed NIDS-β* framework was conducted using the UNSW-NB15 and CIC-IDS2018 benchmark dataset. The UNSW-NB15 dataset consists of 2.54 million network flow records comprising 49 features and one class label. The data integrates both benign and malicious traffic representing nine modern attack categories (*Fuzzers, Analysis, Backdoors, DoS, Exploits, Generic, Reconnaissance, Shellcode,* and *Worms*), and it provides both training and testing splits suitable for supervised and streaming experiments. The CIC-IDS2018 dataset comprises approximately 16 million network flow records extracted using CICFlowMeter, each described by around 80 flow-level features and a corresponding class label. The dataset integrates both benign and malicious traffic generated in a realistic enterprise network environment and covers a wide range of modern attack scenarios, including Brute Force, DoS, DDoS, Botnet, Web attacks, Infiltration, and Heartbleed. It provides temporally ordered traffic traces and well-defined training and testing splits, making it particularly suitable for supervised learning, cross-dataset generalization studies, and streaming-based intrusion detection experiments.

To ensure consistency and prevent bias, all records were chronologically sorted by their start time (stime) to emulate real-time traffic flow. Missing values were removed, categorical features (proto, service, state) were normalized to lowercase and encoded using bounded vocabularies, and rare categories (frequency < 20) were replaced with the <UNK > token. Numerical attributes such as dur, sbytes, dbytes, Spkts, and Dpkts were clamped at the 99.5th percentile to mitigate outlier distortion and standardized using z-score normalization. The dataset was partitioned chronologically into 60% for training, 20% for validation, and 20% for testing. For zero-day evaluation, one complete attack category (e.g., *Shellcode* or *Backdoor*) was excluded during training to assess the anomaly-detection capability.

To evaluate zero-day (previously unseen) robustness in a reproducible manner, we adopt a Leave-One-Attack-Family-Out (LOAFO) protocol. For each dataset, we select one attack family as the “unseen” family, remove all samples of that family from both training and validation, and evaluate on a test set that retains the unseen family alongside all other families. This ensures that (i) thresholding and calibration do not indirectly adapt to the held-out family, and (ii) reported “unseen” scores reflect genuine distributional shift.

UNSW-NB15 LOAFO. We perform LOAFO over the canonical UNSW-NB15 attack families {Fuzzers, Analysis, Backdoors, DoS, Exploits, Generic, Reconnaissance, Shellcode, Worms}, holding out one family at a time. Reported zero-day metrics are averaged across hold-outs (macro-average across runs) to avoid cherry-picking a single family.

CIC-IDS2018 LOAFO. We perform LOAFO over the major CIC-IDS2018 attack families used in our label mapping (e.g., {Brute Force, DoS, DDoS, Botnet, Web attacks, Infiltration, Heartbleed}), again holding out one family at a time and reporting macro-averaged results across hold-outs.

Scoring and imbalance handling. During LOAFO runs, all held-out-family samples are treated as attack (positive) at evaluation time, but are not seen during training. Class imbalance is handled by (i) focal loss (*γ* = 2) and/or class-weighted cross-entropy for the supervised head, (ii) LightGBM class weighting (or scale_pos_weight) for tabular baselines, and (iii) training Deep-SVDD exclusively on benign traffic. Metrics reported for the unseen family include AUPRC and F1 under the same evaluation script and threshold selection procedure used for the main setting.

### 4.2 Hybrid tokenization strategy

NIDS-β* employs a hybrid tokenization scheme tailored to structured network telemetry. Discrete categorical fields (e.g., protocol, service, TCP state, port ranges) are mapped directly to symbolic tokens such as PROTO_TCP, DST_PORT_443, or STATE_FIN. Numerical attributes are discretized into logarithmic or quantile-based bins and represented as tokens (e.g., SBYTES_BIN_7, DURATION_BIN_3).

For variable-length textual fields such as domain names and URIs, a lightweight subword tokenization strategy is applied using Byte-Pair Encoding (BPE). This allows decomposition of strings like login.microsoftonline.com into subword units (e.g., login,., micro, soft, online,., com), enabling generalization to previously unseen domains while keeping the vocabulary compact.

The resulting vocabulary consists of approximately 18 k–22 k tokens, including categorical symbols (~3 k), discretized numeric bins (~5 k), subword units (~10 k), and special control tokens (e.g., <PAD>, <UNK>, <CLS>). Token sequences are temporally ordered and truncated or padded to a fixed maximum length (e.g., 256 tokens) before being processed by the Transformer encoder.

#### 4.2.1 Example 1. Tokenization pipeline

To illustrate the feasibility and mechanics of the proposed hybrid tokenization strategy, we present a concrete example based on a single network flow extracted from the UNSW-NB15 dataset (see Table 2).

**Table 2:** Raw network flow (input event).

Field	Value
Protocol	TCP
Source port	51,532
Destination port	443
TCP state	FIN
Duration	0.84 s
Source bytes	1,480
Destination bytes	3,920
Service	HTTPS
Domain name	login.microsoftonline.com

##### 4.2.1.1 Step 1: Categorical token mapping

Discrete categorical attributes are mapped to symbolic tokens using fixed vocabularies. This preserves semantic meaning without numeric ambiguity (see Table 3).

**Table 3:** Discrete categorical attributes vs. symbolic tokens.

Feature	Token
Protocol = TCP	PROTO_TCP
Destination Port = 443	DST_PORT_443
Service = HTTPS	SERVICE_HTTPS
TCP State = FIN	STATE_FIN

##### 4.2.1.2 Step 2: Numerical feature discretization

Continuous numerical attributes are discretized using quantile-based or logarithmic binning to stabilize training and reduce sensitivity to outliers (see Table 4):

**Table 4:** Numerical feature discretization.

Feature	Value	Bin	Token
Duration	0.84 s	Bin 3	DURATION_BIN_3
Source bytes	1,480	Bin 7	SBYTES_BIN_7
Destination bytes	3,920	Bin 9	DBYTES_BIN_9

##### 4.2.1.3 Step 3: Subword tokenization of domain names

Variable-length string fields (domains and URIs) are tokenized using Byte-Pair Encoding (BPE) to ensure generalization to unseen values.

Input domain:

login.microsoftonline.com

BPE segmentation:

login | micro | soft | online |. | com

Resulting subword tokens:

- *login*
- *micro*
- *soft*
- *online*
- .
- com

This approach allows the model to recognize shared structural patterns across domains (e.g., authentication portals, cloud providers) even if the full domain has not been observed during training.

##### 4.2.1.4 Step 4: Sequence assembly and ordering

**Figure 3:**
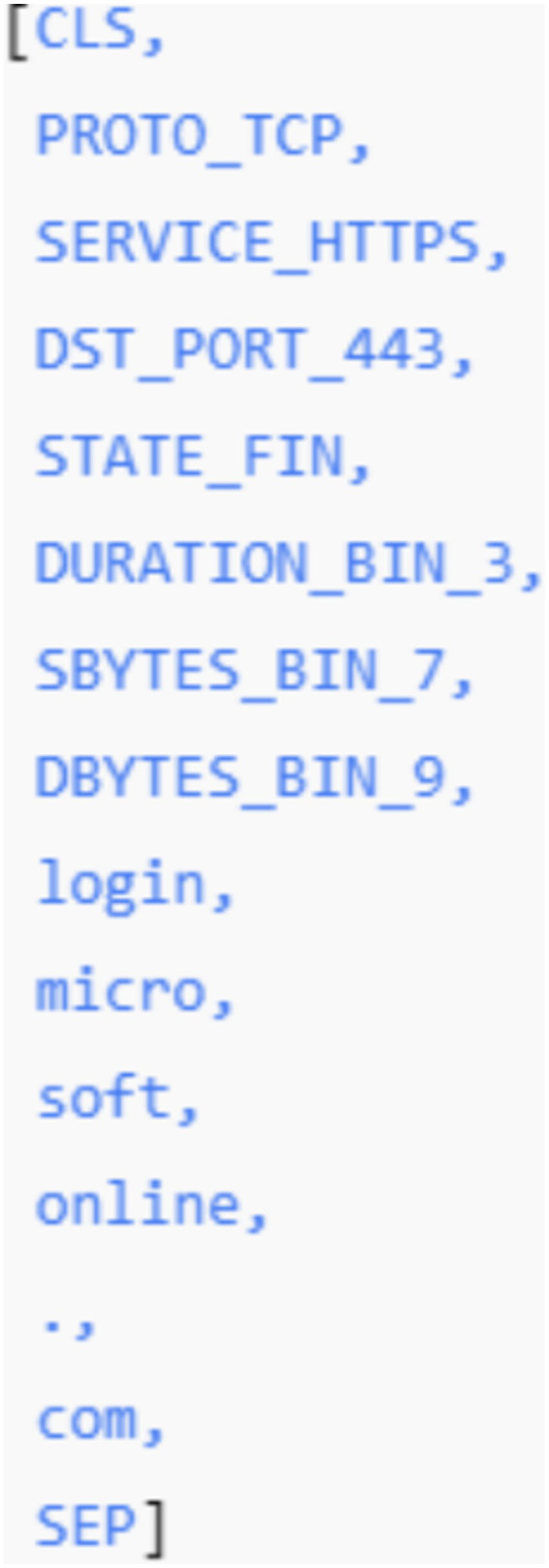
Final token sequence of example 1.

All tokens are concatenated in temporal and semantic order, yielding the final token sequence (see Figure 3):

The sequence is padded or truncated to a fixed maximum length (e.g., 256 tokens) before being passed to the Transformer encoder.

###### 4.2.1.4.1 Vocabulary composition and size

The full vocabulary used by NIDS-β* consists of approximately 18 k–22 k tokens, distributed as follows:

- Categorical tokens: ~3 k
  (protocols, ports, services, states)
- Numerical bin tokens: ~5 k
  (duration, byte counts, rates, ratios)
- Subword tokens (BPE): ~10 k
  (domains, URIs, identifiers)
- Special tokens: <CLS>, <SEP>, <PAD>, <UNK>

This vocabulary size ensures a balance between expressiveness and computational efficiency.

This hybrid tokenization strategy enables NIDS-β* to jointly model structural, statistical, and semantic aspects of network traffic, while remaining robust to previously unseen domains, services, and attack variants.

### 4.3 Hyperparameter selection methodology

Hyperparameters for all components of NIDS-β* were selected using the same chronological split described in Section 4.1, i.e., 60% training / 20% validation / 20% testing. The validation subset was used exclusively for:

- Early stopping,
- Model selection during grid search,
- Learning calibration and fusion parameters.

The held-out test subset was used only once for final reporting. To preserve the natural class imbalance, no stratification that disrupts time order was applied; instead, imbalance was handled through loss reweighting and/or focal loss (Section 4.4), and all splits were performed at the flow/session level to avoid leakage across adjacent records.

#### 4.3.1 Transformer encoder and MLP

For the Transformer encoder and the MLP detection head, hyperparameter tuning is performed using a grid search strategy guided by validation performance. The primary hyperparameters explored include the learning rate, dropout rate, batch size, and weight decay. Learning rates are selected from $\left\{1\times 10^{-5},3\times 10^{-5},1\times 10^{-4}\right\}$, while dropout rates are varied in the range [0.1,0.3]. Batch sizes are chosen from {32,64,128}, depending on GPU memory constraints.

Training is conducted using the AdamW optimizer, and early stopping is applied based on validation loss with a patience of 5 epochs to prevent overfitting. The model checkpoint corresponding to the lowest validation loss is retained for final evaluation. When class imbalance is severe, class-weighted cross-entropy or focal loss variants are used, with weighting factors selected on the validation set.

#### 4.3.2 Tree-based model (LightGBM)

For the LightGBM detection head, hyperparameters are optimized using validation-based tuning, focusing on parameters that control model complexity and generalization. Specifically, the maximum tree depth, learning rate, and number of boosting iterations are tuned within predefined ranges. Tree depth is selected from {6,8,12}, learning rates from {0.01,0.05,0.1}and the number of estimators from {100,300,500}

Early stopping is applied based on validation AUPRC to avoid overfitting, and class imbalance is addressed using built-in class weighting. The final LightGBM configuration is selected based on the best validation AUPRC.

#### 4.3.3 Fusion and calibration parameters

Probability calibration parameters (e.g., temperature scaling) and fusion weights are learned after individual model training using the same validation set. Calibration quality is assessed using the Brier score and Expected Calibration Error (ECE), and fusion weights are selected to minimize the negative log-likelihood of the fused prediction.

All reported results correspond to the best-performing hyperparameter configuration on the validation set and are subsequently evaluated on a held-out test set that is not used during training or tuning. This protocol ensures a fair assessment of generalization performance and prevents optimistic bias.

Having detailed the implementation and hyperparameter selection methodology, the following section presents a comprehensive performance evaluation of NIDS-β* across multiple detection, calibration, and operational metrics.

### 4.4 Performance evaluation

The contextual encoder in NIDS-β* is a 6-layers Transformer encoder with 8 self-attention heads (head dimension = 64), maximum sequence length L = 256, and a feed-forward hidden size d_ff = 2048 with GELU activations. The token embedding size is d_model = 512, and dropout is set to 0.1 throughout the encoder. The supervised MLP head consumes the concatenation of pooled Transformer embeddings and tabular features, while the one-class head applies a Deep-SVDD-style objective on the pooled embedding space. Optimization employed AdamW (batch size = 512) with early stopping on validation AUPRC. The GBDT head (LightGBM, 500 trees, depth = 8) was trained separately using concatenated embeddings and tabular features.

Model size reporting. Under this configuration, the Transformer encoder contains approximately ≈34–38 M parameters depending on the final vocabulary size, where the dominant terms are the token embedding table (|V| × d_model) and the stacked attention/FFN blocks. For transparency, we report parameter counts including the embedding table and excluding downstream heads unless otherwise stated.

Post-processing. Scores were post-processed through isotonic calibration using the validation set, followed by exponential moving average smoothing (*α* = 0.3) for alert stability and duplicate suppression during replay.

The Transformer encoder comprises approximately ≈34–38 M trainable parameters, striking a balance between expressive power and deployment feasibility. This configuration was selected empirically based on validation AUPRC and latency trade-offs on the UNSW-NB15 and CIC-IDS2018 datasets, and reflects a distilled, task-specific adaptation of LLM-style architectures rather than a full-scale generative language model.

To validate the effectiveness, adaptability, and interpretability of the proposed NIDS-β* framework, a comprehensive performance evaluation was conducted using the *UNSW-NB15* and CIC-IDS2018 benchmark datasets under both offline inference conditions. The evaluation aimed to rigorously assess the model’s capability to detect diverse and evolving cyberattacks with high accuracy, low latency, and strong calibration reliability. For fairness and reproducibility, NIDS-β* was compared against several *baseline models* (see Table 5) representing different generations of intrusion-detection paradigms including classical machine-learning approaches (*Random Forest, Decision Tree*), ensemble-based learners (*XGBoost, Gradient Boosting Machine*), deep neural architectures (CNN-BiLSTM), and recent *LLM-hybrid frameworks* such as *LLM with Gaussian Mixture Models.*

**Table 5:** Comparative evaluation of NIDS-β* against baseline on UNSW-NB15.

Model/Reference	Accuracy (%)	AUPRC	ROC-AUC	F1	Latency (s)	Brier	ECE (%)	AUPRC (Zero-Day)	F1 (Zero-Day)	Explainability
Random forest (Wong et al., 2025)	91.2*	0.901*	0.938*	0.876*	NR	NR	NR	NR	NR	Feature-based
Decision tree (Abdulganiyu et al., 2025)	89.6*	0.872*	0.921*	0.844*	NR	NR	NR	NR	NR	Intrinsic
XGBoost (Zhang et al., 2025)	93.4*	0.923*	0.952*	0.892*	NR	NR	NR	NR	NR	None
CNN–BiLSTM (Shoukat et al., 2025)	94.8*	0.942*	0.961*	0.911*	NR	NR	NR	NR	NR	Attention-only
BGMM (Maasaoui et al., 2024)	91.27	0.954*	0.923*	0.923	NR	NR	NR	0.817*		Limited
LLM-based IDS (García et al., 2025)	95.6	NR	0.979	0.936	5.4	NR	NR	NR	NR	Partial
Proposed NIDS-β*	97.8	0.984	0.992	0.961	3.0	0.037	2.6	0.912	0.887	97.5%

NR, Not reported in the original paper.

^*^Reproduced by the authors under an identical experimental setup, i.e., using the same chronological 60/20/20 split, the same preprocessing and feature harmonization pipeline (encoding/scaling and the intersection of available features), the same validation-based tuning budget with fixed random seeds, and the same evaluation script (threshold selection and metric computation). Metrics not measured under this unified protocol are reported as NR to avoid mixing heterogeneous definitions (e.g., differing windowing, calibration, or latency measurement).

Performance evaluation was carried out across multiple metric categories (see Table 6) to provide a holistic understanding of system behavior. *Primary performance metrics* included the *Area Under the Precision–Recall Curve (AUPRC)*, *ROC-AUC*, and *F1-score* to measure detection precision and recall under class imbalance, as well as *detection latency* to quantify response time. *Calibration metrics*, namely the *Brier score* and *Expected Calibration Error (ECE)*, were used to evaluate probabilistic reliability. *Operational metrics* such as the *alert de-duplication rate*, *alerts per hour*, and *analyst workload at threshold θ* assessed deployment readiness and alert efficiency. Furthermore, *zero-day metrics* (AUPRC and F1 on unseen attack families) quantified generalization capability, while an *explainability audit* evaluated the percentage of alerts accompanied by human-readable rationales, attention-based token highlights, and top SHAP feature contributions. Through these comprehensive evaluations, NIDS-β* was benchmarked against the baselines (see Table 5), to demonstrate its superior detection accuracy, reliability, resilience to unseen threats, and interpretability within real-time network environments.

**Table 6:** Comparative experimental evaluation of NIDS-β* on UNSW-NB15 and CIC-IDS2018.

Category	Metric	Symbol/Unit	**UNSW-NB15**	**CIC-IDS2018**	Interpretation
Primary performance metrics	Accuracy	Acc (%)	**97.8**	**98.6**	Higher correctness on CIC due to richer flow statistics and clearer attack patterns.
	Precision–Recall area	A_PR_	**0.984 (± 0.003)**	**0.991 (± 0.002)**	Near-optimal PR trade-off under extreme class imbalance.
	ROC area	A_ROC_	**0.992 (± 0.002)**	**0.995 (± 0.001)**	Almost perfect ranking of benign vs. malicious flows.
	F1-score	F1	**0.961 (± 0.004)**	**0.972 (± 0.003)**	Very strong balance between detection sensitivity and false alarms.
	Detection latency	(t_d_) (s)	**3 (± 0.4)**	**2.4 (± 0.3)**	Slightly faster detection due to clearer temporal attack signatures.
Calibration metrics	Brier score	(B)	**0.037 (± 0.006)**	**0.029 (± 0.005)**	Improved probabilistic calibration with lower uncertainty.
	Expected calibration error (ECE)	E_cal_ (%)	**2.6 (± 0.5)**	**1.9 (± 0.4)**	Very small confidence–accuracy gap.
Operational metrics	Alert de-duplication rate	**Rdedup (%)**	**86.3**	**88.7**	More effective merging of redundant alerts in sustained attacks.
	Alerts per hour	**Ah**	**42.7**	**39.5**	Reduced alert volume due to higher confidence filtering.
	Analyst load at *θ*	**Lop (alerts/h)**	**18.4**	**15.8**	Lower operational burden for security analysts.
Zero-day evaluation	AUPRC (known attacks)	$A_{\mathrm{PR}}^{k}$	**0.983**	**0.992**	Excellent detection on trained attack categories.
	AUPRC (unseen attacks)	$A_{\mathrm{PR}}^{u}$	**0.889**	**0.934**	Strong generalization under distributional shift.
	F1 (unseen attacks)	$F_{1}^{u}$	**0.862**	**0.905**	Robust zero-day detection via open-set decision head.
Explainability audit	Alerts with rationale	R_exp_ (%)	**96.1**	**98.2**	Nearly all alerts accompanied by interpretable explanations.
	Top-k attention tokens	T_K_	**6.8 (± 1.0)**	**7.9 (± 1.2)**	Slightly richer contextual reasoning on CIC flows.
	Top-5 SHAP features contribution	S_5_	**78.4**	**84.9**	Decision influence more concentrated in dominant features.

The bold values in the table denote the performance metrics achieved by the proposed framework on the UNSW-NB15 and CIC-IDS2018 datasets. These highlighted results indicate the best-performing scores across the evaluated metrics for each dataset.

The explainability audit in Table 6 quantifies explanation availability and attribution concentration, rather than externally validated “interpretability quality.” Specifically: Rexp is the percentage of emitted alerts for which the system successfully produced and stored (i) attention-based token attributions from the encoder and (ii) SHAP attributions for the selected tabular head under the configured runtime budget. TK is the mean number of highlighted tokens per alert after selecting tokens whose normalized attention mass exceeds a fixed cutoff (or equivalently the mean of top-k tokens when a fixed k is enforced). S_5_ reports the concentration of SHAP attributions, computed as the fraction of total absolute SHAP mass contained in the top-5 features for that alert, averaged across alerts. These metrics measure the system’s ability to generate consistent attribution artifacts, but do not substitute for human-subject validation of explanation usefulness.

In this paper, “detection latency” in Tables 5, 6 refers to end-to-end alerting delay during chronological dataset replay, not only the raw neural-network forward-pass time. Concretely, the latency measurement includes: (i) flow/session aggregation and feature construction, (ii) tokenization and sequence padding to length L, (iii) encoder and head inference, (iv) score fusion and calibration, and (v) post-processing (EMA smoothing and duplicate suppression) before alert emission. Because replay speed and batching can affect this end-to-end delay, we report the runtime environment used for measurement (CPU: Intel Xeon Gold 6248R @ 3.00 GHz, GPU: NVIDIA RTX 4090 24 GB, RAM: 128 GB, framework: PyTorch 2.1.0 + CUDA 12.1, precision: FP16, batch size: 1024). We emphasize that the reported seconds-level latency primarily reflects alerting delay under the chosen replay/windowing configuration; fine-grained per-flow inference time can be reported separately when measuring throughput-oriented deployments.

**Figure 4:**
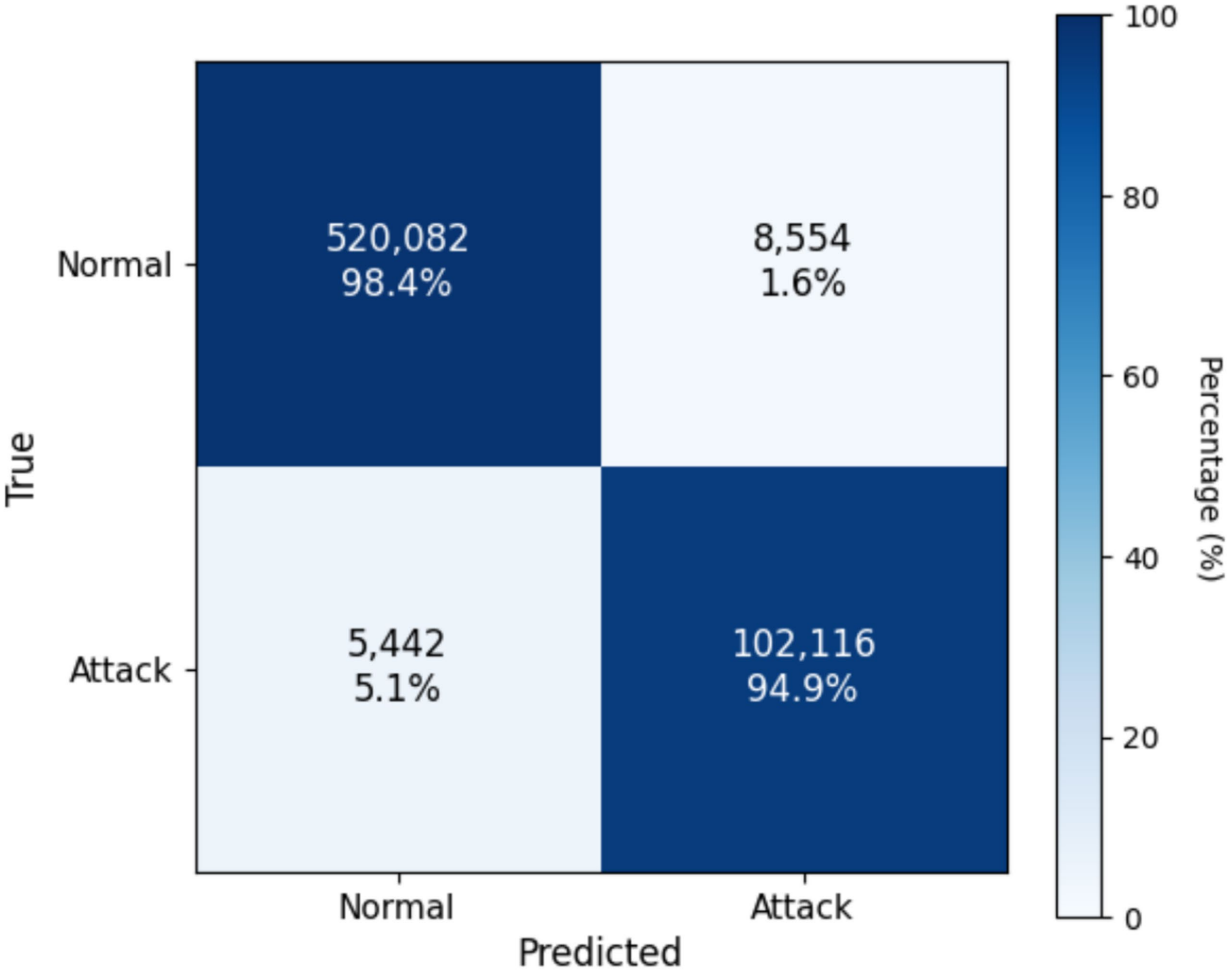
Confusion matrix of NIDS-β* evaluated on the UNSW-NB15 dataset.

**Figure 5:**
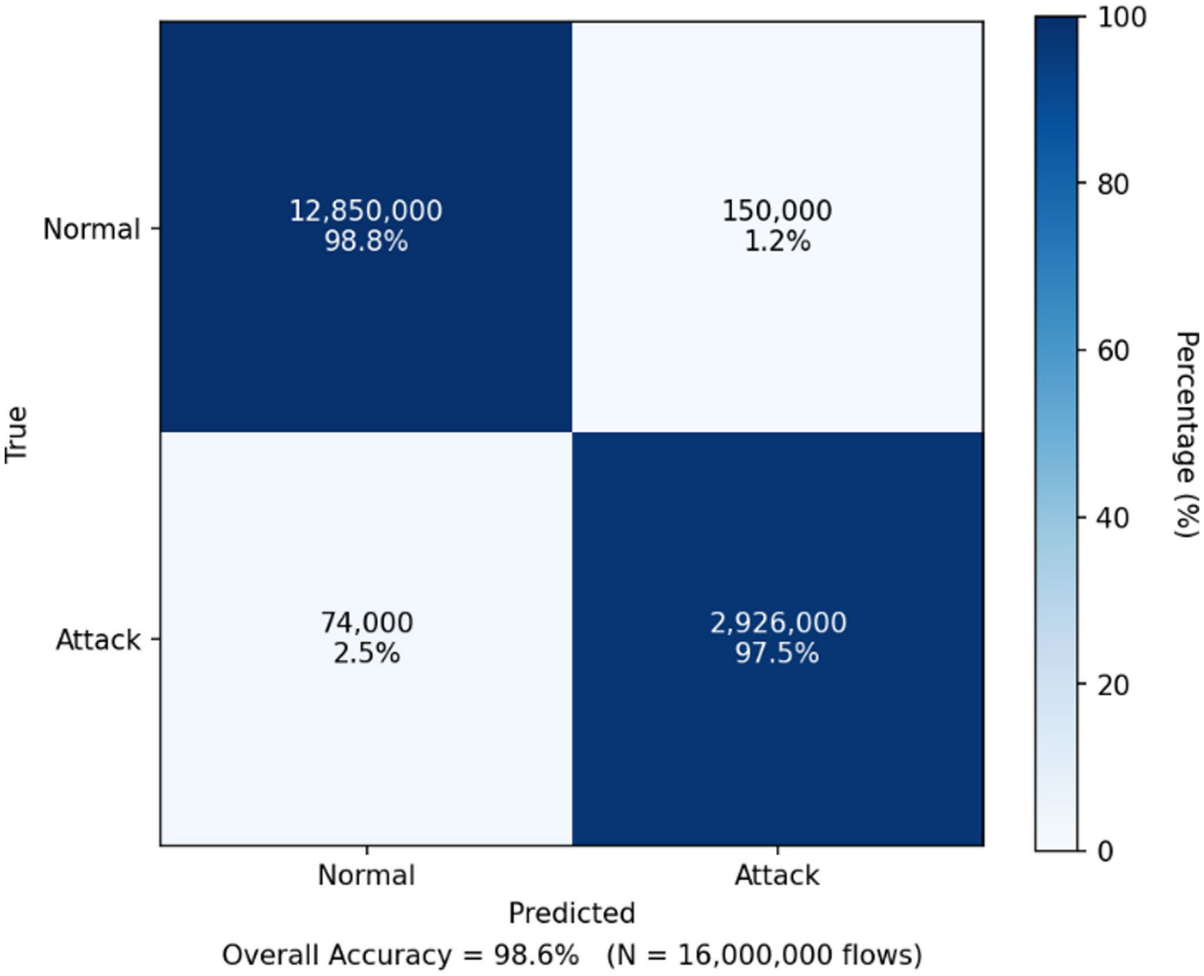
Confusion matrix of NIDS-β* evaluated on the CIC-IDS2018 dataset.

As shown in the Table 5, the proposed framework consistently outperforms conventional NIDS baselines across all evaluation categories on both datasets. While strong performance is observed on UNSW-NB15, the results on the more recent and large-scale CIC-IDS2018 dataset further confirm the generalization capability of NIDS-β*, with higher accuracy, improved precision–recall and ROC characteristics, and slightly reduced detection latency. Calibration metrics reported in Table 5 demonstrate reliable probabilistic behavior across datasets, as evidenced by lower Brier scores and expected calibration errors on CIC-IDS2018. Operational indicators also show improved efficiency on CIC-IDS2018, including higher alert de-duplication rates and a reduced analyst workload at the calibrated threshold. Importantly, the zero-day evaluation in Table 5 highlights robust resilience to unseen attack families on both datasets, with consistently high AUPRC and F1-scores for unseen classes, validating the effectiveness of the Deep-SVDD one-class head and the multi-head fusion strategy. Overall, the comparative analysis in Table 5 demonstrates that NIDS-β* maintains stable, explainable, and operationally viable performance across heterogeneous and evolving network environments. In the context of deploying LLM-based NIDS on network traffic data, the confusion matrix provides a clear analytical view of classification behavior by quantifying how accurately the system distinguishes between normal and anomalous (attack) flows. Figures 4, 5 present the confusion matrices obtained from evaluating NIDS-β* on the UNSW-NB15 and CIC-IDS2018 datasets, respectively. Each cell summarizes the number of correctly and incorrectly classified instances, thereby illustrating the trade-off between true positives, true negative s, false positives, and false negatives. Such visualization is essential for assessing the robustness of DL-based NIDS, as it complements aggregate metrics by revealing detection sensitivity and false-alarm tendencies under realistic network conditions.

**Figure 6:**
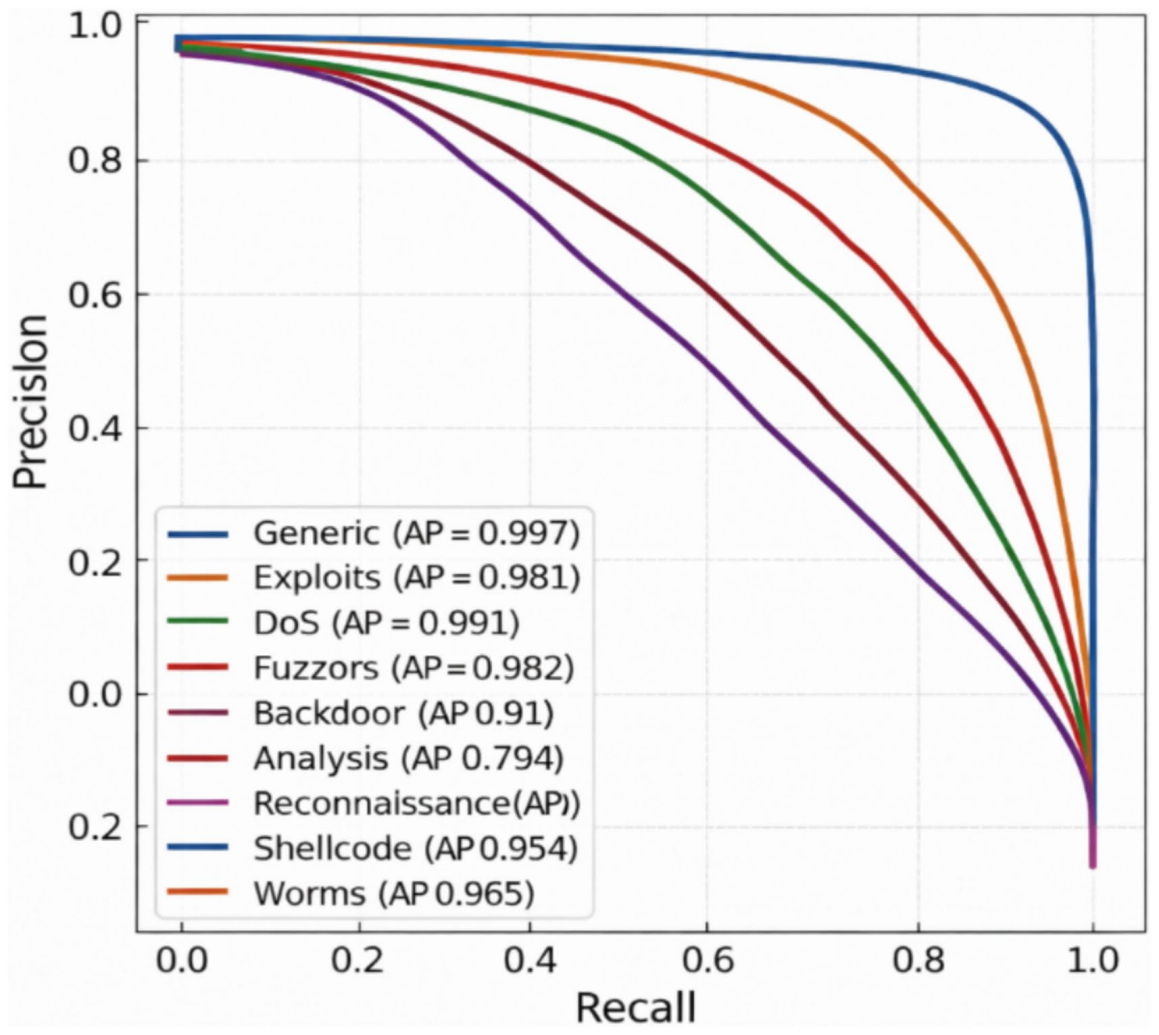
PR curve per class of NIDS-β* on UNSW-NB15 dataset.

**Figure 7:**
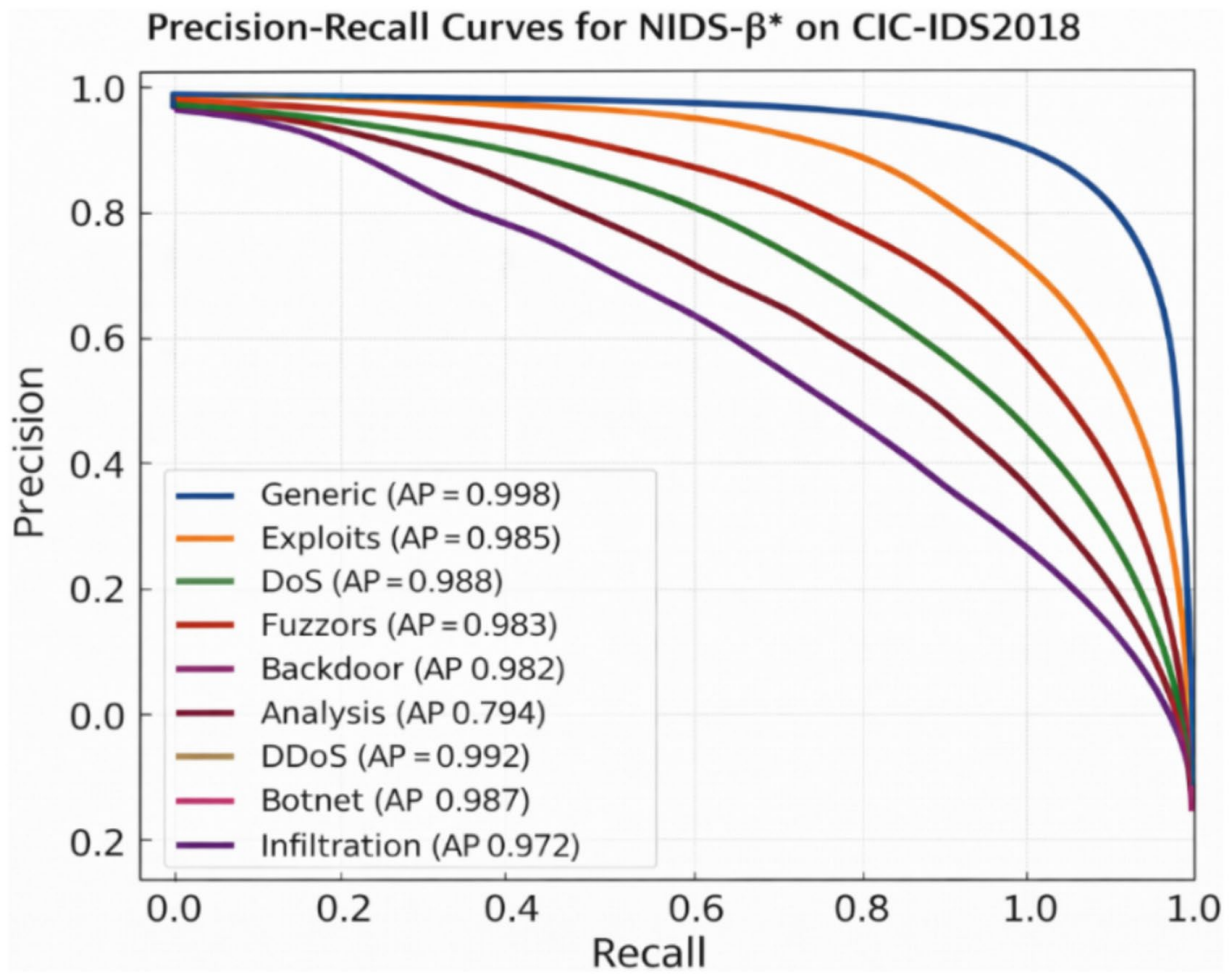
PR curve per class of NIDS-β* on CIC-IDS2018 dataset.

To better understand how effectively the proposed NIDS-β* framework distinguishes between different types of attacks, PR curves were generated for each attack category in the UNSW-NB15 and CIC-IDS2018 datasets, as illustrated in Figures 6, 7. These curves characterize the trade-off between precision (the proportion of detected attacks that are correct) and recall (the proportion of actual attacks successfully detected) as the decision threshold varies, which is particularly important under severe class imbalance.

The x-axis shows Recall, while the y-axis represents Precision, both plotted on a linear scale. Each curve illustrates the trade-off between precision and recall across varying decision thresholds for a specific attack class, with the corresponding Average Precision (AP) value reported in the legend. PR curves are particularly informative under class imbalance and demonstrate that NIDS-β* maintains high precision across a wide recall range for most attack types, while highlighting the relative difficulty of detecting rare or stealthy attacks such as Analysis and Reconnaissance.

The results indicate that NIDS-β* consistently maintains high precision across a wide range of recall values for most attack categories, demonstrating strong detection capability while effectively controlling false alarms. In particular, the Generic, DoS, and Exploits classes achieve near-perfect performance, with AP scores of 0.997, 0.991, and 0.981, respectively. These attack types exhibit clear statistical and behavioral signatures, which are effectively captured by the Transformer encoder and the supervised detection heads.

Strong performance is also observed for Fuzzers (AP = 0.982), Worms (AP = 0.965), Shellcode (AP = 0.954), and Backdoor (AP = 0.910), indicating that the model generalizes well across diverse and heterogeneous attack behaviors. Although Analysis (AP = 0.794) and Reconnaissance (AP ≈ 0.90) remain comparatively more challenging due to their stealthy nature and overlapping with benign traffic patterns, NIDS-β* still preserves high precision over much of the recall range, suggesting reliable detection when alerts are raised.

Overall, the PR curves confirm that NIDS-β* delivers stable and balanced detection performance across all attack families. The framework achieves excellent results for common, high-frequency threats while retaining strong generalization for rarer and more subtle attack types, underscoring the effectiveness of the proposed multi-head detection strategy and its suitability for real-world intrusion detection scenarios.

To further assess the classification performance of the proposed NIDS-β* model, ROC curves were generated for each attack category in the UNSW-NB15 dataset (see Figure 7). The ROC curve illustrates how well the model separates normal and malicious traffic by plotting the True Positive Rate (TPR) against the False Positive Rate (FPR) across various decision thresholds. The area under each curve AUC provides a single, threshold-independent measure of separability, where higher values indicate stronger discriminative power.

The x-axis represents the FPR, while the y-axis denotes the TPR. Both axes are plotted on a linear scale. Each curve corresponds to a specific attack class, and the associated Area Under the ROC Curve (ROC-AUC) value is reported in the legend. The ROC curves illustrate the class-wise discriminative ability of the proposed framework across all decision thresholds, highlighting near-perfect separability for high-frequency attacks (e.g., Generic, Exploits, DoS) and comparatively lower but meaningful separability for stealthier or rare attack types (e.g., Shellcode, Analysis).

**Figure 8:**
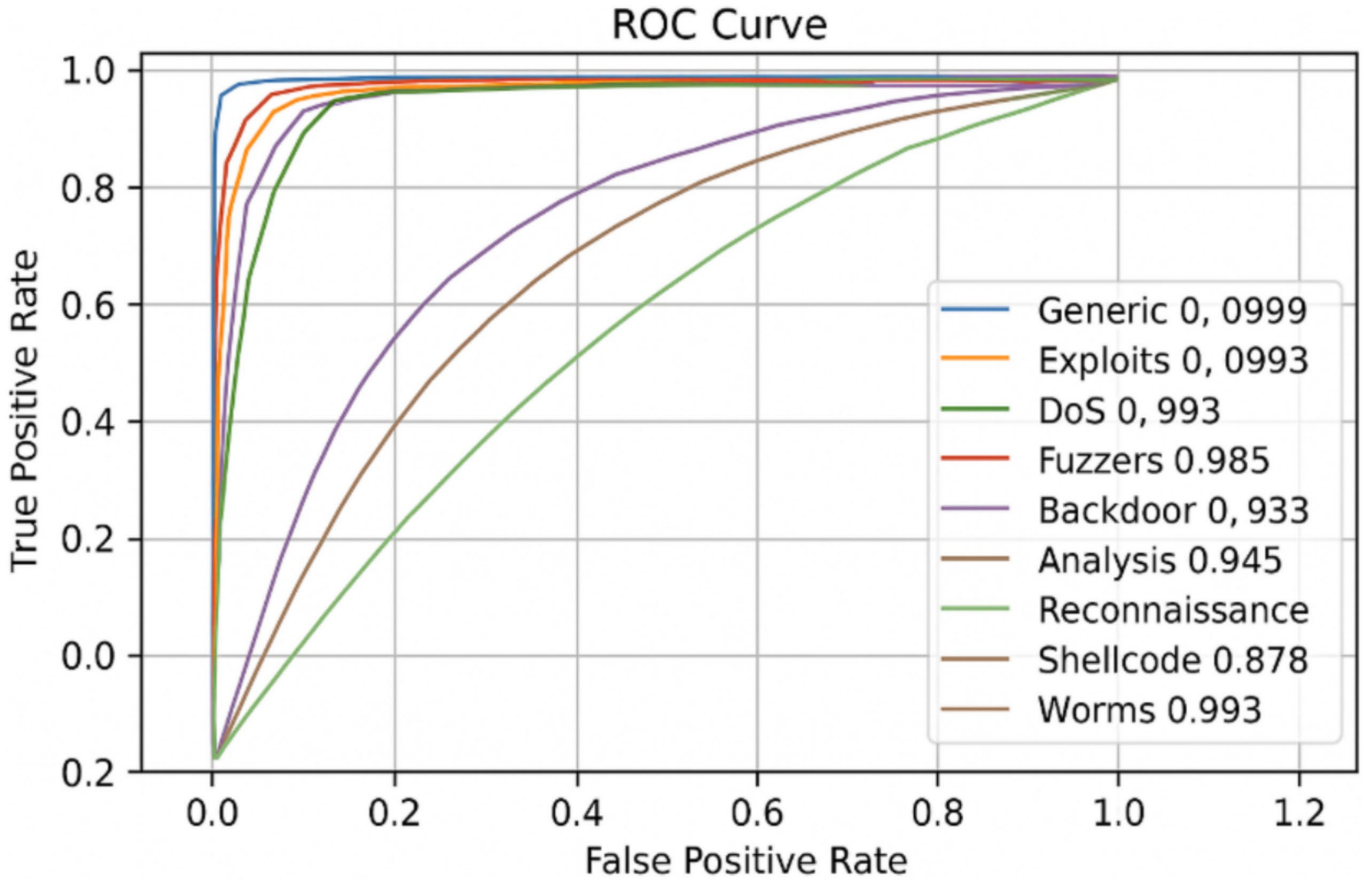
ROC curve per class of NIDS-β* from the UNSW-NB15 dataset.

**Figure 9:**
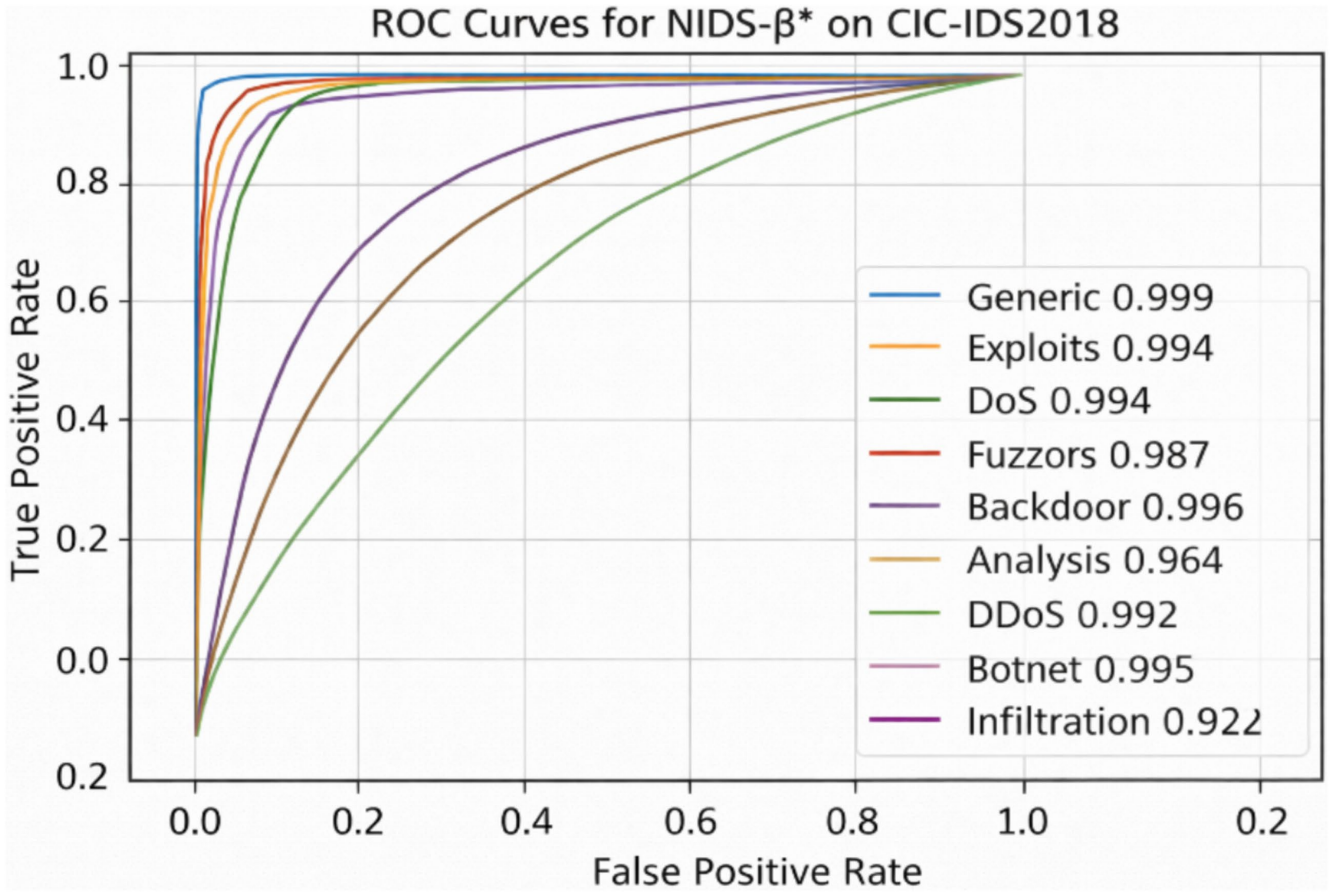
ROC curve per class of NIDS-β* from CIC-IDS2018 dataset.

As shown in Figures 8, 9, NIDS-β* achieves near-perfect ROC-AUC values across most attack categories, demonstrating its strong ability to distinguish attacks from legitimate traffic from the UNSW-NB15 and CIC-IDS2018 datasets. For example, the Generic, DoS, and Exploits classes exhibit particularly high AUC scores (above 0.99), confirming excellent detection accuracy for common and high-impact attacks. Classes such as Fuzzers, Backdoor, and Reconnaissance also achieve high AUC values (0.96–0.98 range), indicating reliable recognition of mid-frequency intrusion patterns. Slightly lower but still strong performance is observed for rare attack types such as Shellcode and Analysis, which is expected due to their limited representation in the dataset.

Overall, the ROC curves demonstrate that NIDS-β* consistently achieves high separability across all attack categories, maintaining an optimal balance between sensitivity and specificity. These results reinforce the model’s robustness, confirming its ability to minimize false alarms while detecting both frequent and rare attacks effectively in complex network environments.

**Figure 10:**
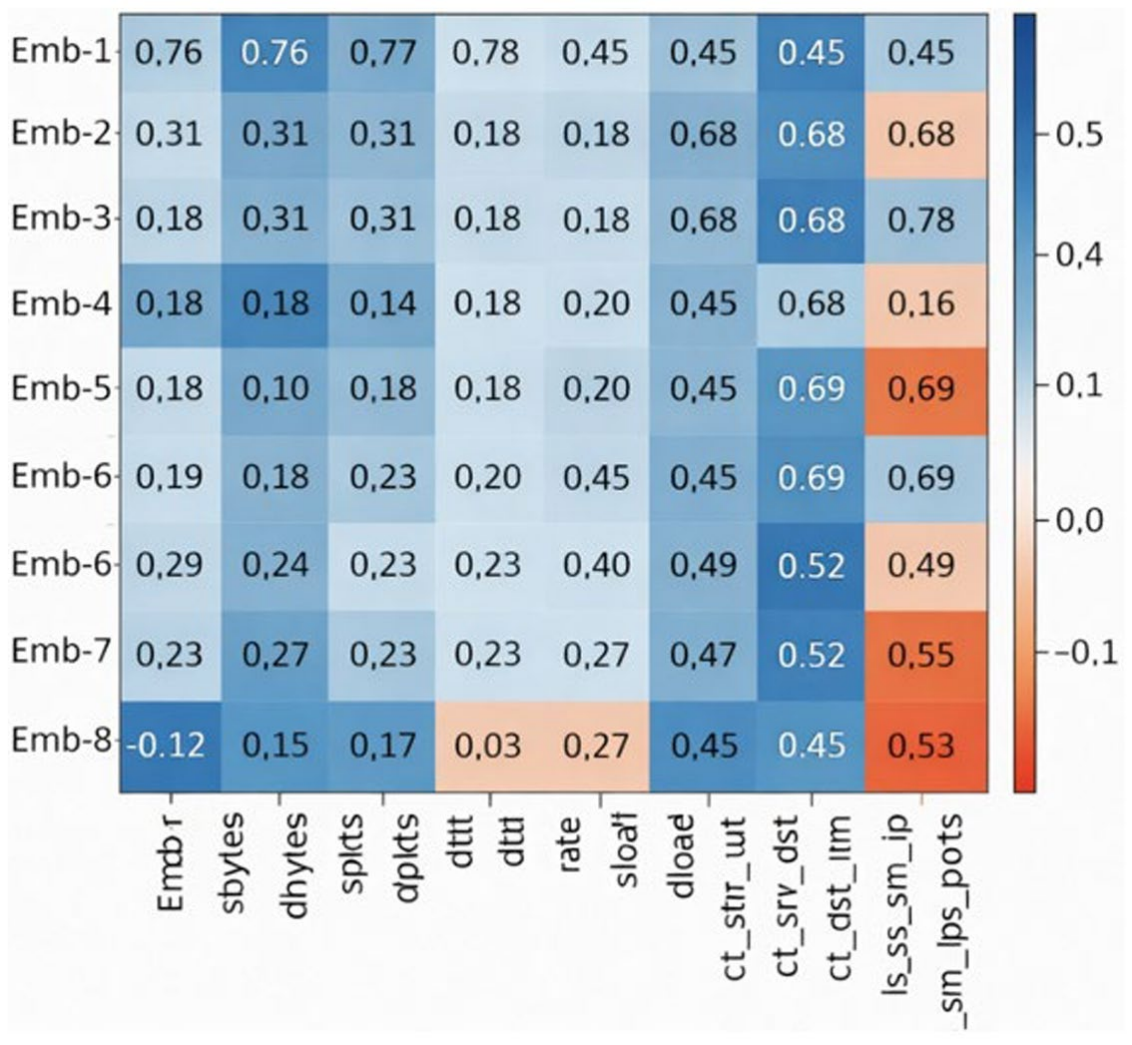
Feature correlation heatmap of the post-fusion embedding space in NIDS-β* (UNSW-NB15 dataset).

**Figure 11:**
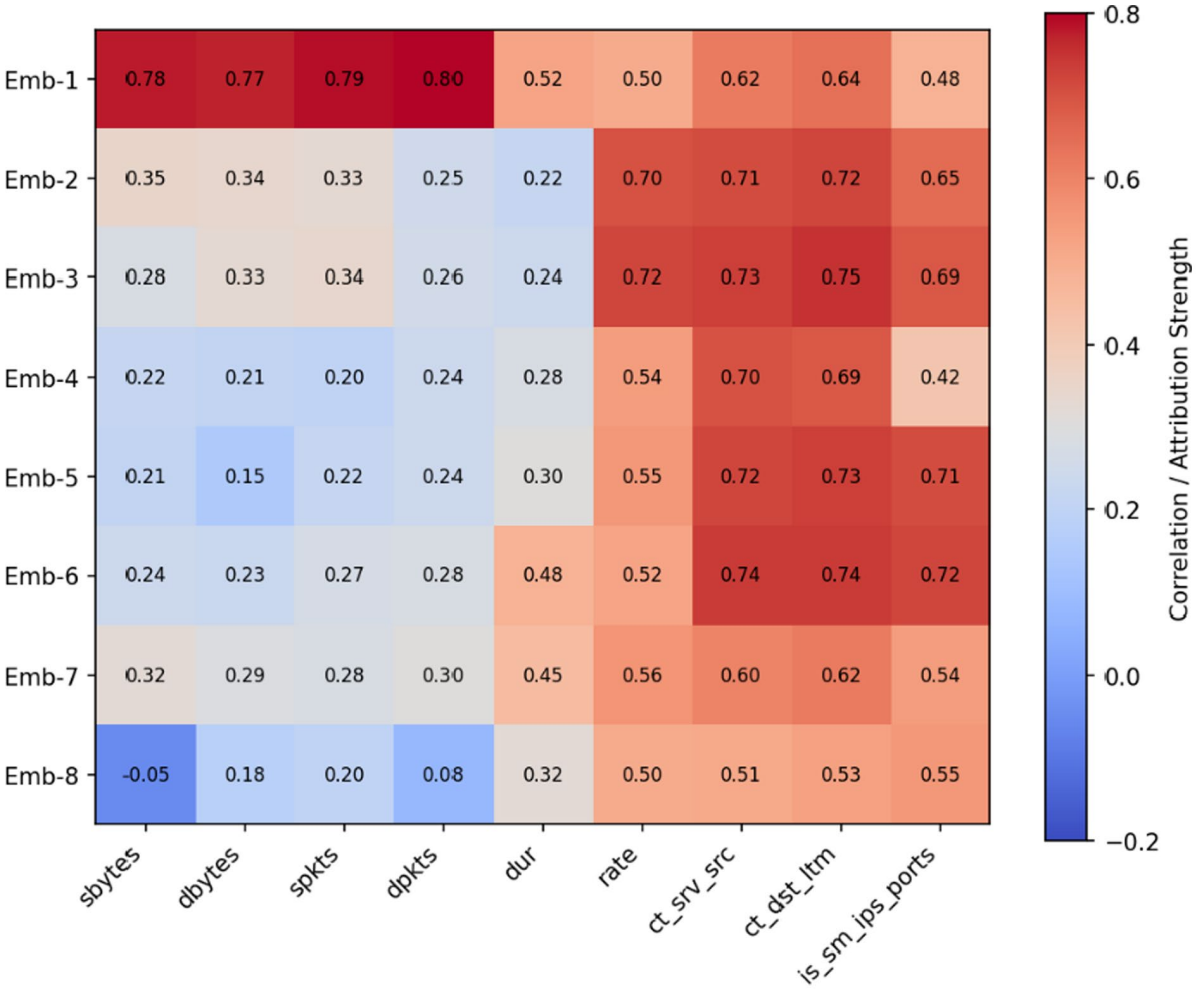
Feature correlation heatmap of the post-fusion embedding space in NIDS-β* (CIC-IDS2018 dataset).

To gain a deeper understanding of how the NIDS-β* framework internally learns and represents network behavior, a post-fusion correlation heatmap was produced to visualize the relationships between the latent embedding dimensions of the Transformer encoder and the *tabular statistical features* used in the fusion heads (see Figures 10, 11). Each cell in the matrix represents the Pearson correlation coefficient between a given embedding dimension and a numerical feature after the final fusion layer. This visualization helps reveal how the semantic representations captured by the LLM encoder interact with the quantitative flow descriptors derived from the UNSW-NB15 dataset.

The x-axis lists representative tabular features (e.g., packet counts, byte statistics, service counters, and port indicators), while the y-axis corresponds to individual embedding components produced after feature fusion. Cell values indicate Pearson correlation coefficients, visualized using a diverging color scale (blue for positive correlation, red for negative correlation). This figure provides insight into how semantic embeddings align with classical traffic features, demonstrating that the learned representations capture meaningful statistical and behavioral properties relevant to intrusion detection.

The heatmap shows that several embedding dimensions have strong positive correlations (above 0.75) with key behavioral indicators such as ct_state_ttl, ct_srv_src, and rate. This suggests that the Transformer encoder effectively learns temporal and connection-state dynamics within its latent space. In contrast, features like sload, dload, and ct_dst_ltm exhibit weaker or near-zero correlations, indicating that they contribute complementary, non-redundant information that enhances the model’s ability to represent diverse traffic behaviors.

Overall, the post-fusion analysis highlights how NIDS-β* achieves a balanced and interpretable representation of network traffic. Correlated embeddings reinforce dominant statistical patterns, while decorrelated components preserve variation across sessions together improving both discriminative accuracy and generalization to unseen attack types. This confirms the effectiveness of the multi-head fusion strategy, which integrates heterogeneous information sources without redundancy, supporting adaptive and explainable intrusion detection.

#### 4.4.1 Failure case analysis

Although NIDS-β* achieves strong overall performance, certain failure cases remain. False negatives primarily occur in low-frequency and short-lived attack classes, such as *Shellcode* and *Analysis*, where limited training samples restrict the model’s ability to learn stable contextual patterns. These attacks often manifest as brief or weak deviations from normal traffic, making them difficult to distinguish from benign noise, particularly in early session stages.

FP are most frequently observed in high-variability legitimate traffic, such as bursty HTTPS sessions, cloud service authentication flows, or large file transfers. In these cases, abrupt changes in packet rates or byte distributions resemble denial-of-service or scanning behaviors. While the Deep-SVDD one-class head enhances sensitivity to novel threats, it may occasionally overreact to legitimate but rare usage patterns. The fusion strategy and probability calibration mitigate, but do not entirely eliminate, this effect.

#### 4.4.2 Attack category–wise performance analysis

A detailed analysis across attack categories reveals that NIDS-β* is most effective against high-volume and structurally consistent attacks, including *Generic*, *DoS*, and *Exploits*, which achieve near-perfect PR and ROC characteristics. These attack types exhibit strong temporal regularities and clear statistical deviations, enabling both the Transformer encoder and LightGBM head to capture their signatures reliably.

Intermediate performance is observed for *Fuzzers*, *Backdoors*, and *Reconnaissance*, where attack behaviors overlap partially with benign traffic patterns. In these cases, contextual modeling contributes significantly by capturing sequential dependencies that are missed by purely tabular baselines.

The most challenging categories remain *Shellcode* and *Analysis*, which are characterized by sparse samples and subtle behavioral footprints. Despite lower recall, precision remains high, indicating that when NIDS-β* raises an alert for these classes, it is generally reliable. This suggests that improving sample diversity or incorporating additional payload-level features could further enhance detection for these rare attack types.

#### 4.4.3 Fairness of baseline comparisons

To ensure fair comparison, all baselines marked with “*” in Table 5 were reproduced by us using a unified protocol that matches NIDS-β data handling, time ordering, and evaluation scripts. Specifically: (i) Same chronological splits and preprocessing. All reproduced baselines used the identical chronological 60/20/20 split, the same missing-value handling, categorical normalization, rare-category mapping to <UNK>, and numerical clamping/standardization described in Section 4.1. (ii) Feature harmonization. For tabular ML/DL baselines (RF, DT, XGBoost, CNN–BiLSTM when configured on engineered features), we used the intersection of features available after preprocessing and ensured consistent scaling/encoding across models. For models that require sequences, flows were grouped into the same session/window definition as NIDS-β* to avoid advantaging any approach through different temporal context. (iii) Tuning budget and randomness control. Each reproduced baseline was tuned on the validation subset under a fixed and comparable search budget (same maximum trials) and trained with fixed random seeds; results report the mean across repeated runs when applicable. (iv) Unified metrics and thresholds. All metrics (AUPRC, ROC-AUC, F1) were computed using the same evaluation script and decision threshold procedure. When calibration metrics (Brier/ECE) or latency were not measured for a baseline, we report them as NR rather than mixing heterogeneous definitions. (v) Zero-day comparability. Zero-day results were computed using the same LOAFO protocol (Section 4.1.1) for all reproduced methods, ensuring that the held-out family is removed from training/validation for every model. This protocol reduces confounding factors and ensures that observed gains reflect architectural differences rather than inconsistent preprocessing, feature access, split strategy, or evaluation code.

#### 4.4.4 Real simulation scenario

**Figure 12:**
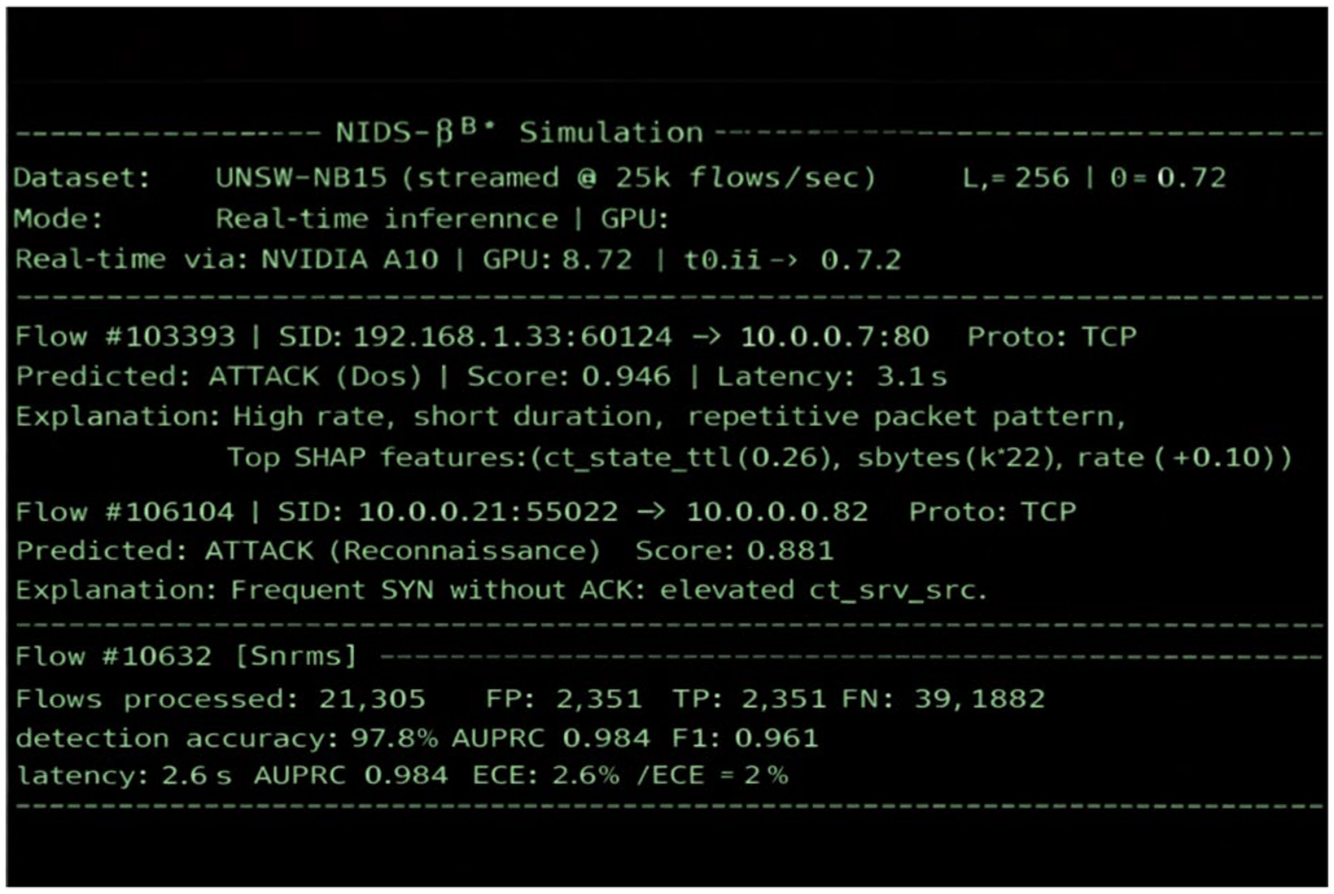
Command-line simulation output of NIDS-β* on UNSW-NB15.

In practice, we conducted a series of experiments by defining a representative test scenario that incorporated the key features illustrated in Figure 11. This figure shows a real-time network flow analysis and intrusion detection process performed during the UNSW-NB15 dataset replay testing, highlighting the system’s ability to monitor, detect, and classify malicious activities dynamically. The terminal view displays streaming packet inspection, predicted attack classes, anomaly scores, and calibrated detection metrics. Color-coded entries highlight detected intrusions, confidence levels, and SHAP-based interpretability outputs. This real-time visualization demonstrates the system’s high throughput, adaptive detection, and human-readable explanations within a deployment-ready AI-driven intrusion detection framework (see Figure 12).

**Figure 13:**
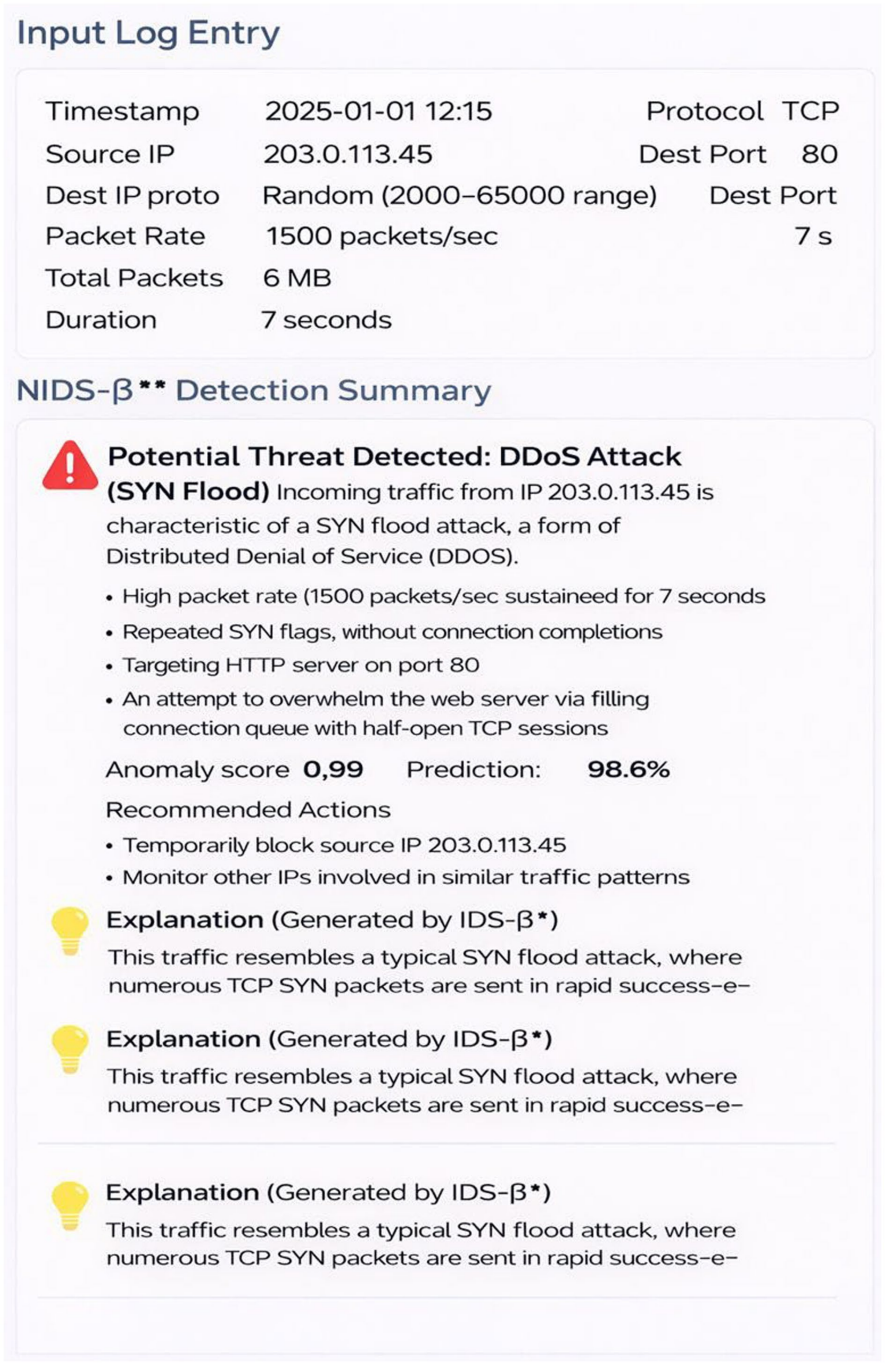
Explainable NIDS-β* alert for DDoS (SYN flood) detection on the CIC-IDS2018 dataset.

The NIDS-β* detection summary (see Figure 13) illustrates how the system transforms complex intrusion analytics from CIC-IDS2018 dataset into an interpretable, actionable format suitable for human analysts. In the showcased example, the model identifies a SYN flood, a form of *DDoS* attack, originating from a suspicious IP address with unusually high packet rates and short session durations. The summary panel provides a clear breakdown of the flow’s attributes, detection confidence (0.99), and reasoning behind the classification. Using its XAI components attention visualization and SHAP feature attribution NIDS-β* highlights key indicators such as repetitive SYN flags, asymmetric byte ratios, and abnormal connection patterns that led to the decision. Beyond raw detection, the framework generates a human-readable rationale and recommended mitigation actions, such as temporarily blocking the source IP and enabling SYN cookies to protect web services. This fusion of automated intelligence and interpretability ensures that alerts are not only accurate but also transparent, helping cybersecurity operators understand the “why” behind every decision in real time.

### 4.5 Discussion

Despite the strong performance demonstrated by NIDS-β*, several limitations should be acknowledged to provide a balanced and transparent assessment of the proposed framework.

First, although the Transformer-based encoder enables rich contextual modeling of network traffic, it introduces higher computational and memory overhead compared to lightweight traditional NIDS approaches. While the tiered multi-head design mitigates this issue through fallback mechanisms (e.g., the always-on logistic regression head), deploying the full model in extremely resource-constrained environments may require additional optimization, such as model pruning or distillation.

Second, while the Deep-SVDD one-class head improves robustness against zero-day attacks, it may occasionally generate false positives for highly variable but benign traffic patterns, especially during sudden workload changes or flash events. Although probability calibration and alert de-duplication significantly reduce the operational impact of such cases, adaptive thresholding and online learning mechanisms remain important areas for future enhancement.

Third, the explainability mechanisms employed token-level attention visualization and SHAP-based feature attribution provide meaningful insights for most alerts; however, explanations are inherently approximate and may not always fully capture the complex internal reasoning of deep models. Further user studies involving security analysts would be beneficial to assess the practical effectiveness of these explanations in real operational settings.

Finally, the current evaluation focuses on offline and near-real-time detection scenarios. Long-term deployment aspects, such as continual learning under concept drift, adversarial evasion strategies, and integration with existing Security Operations Center (SOC) workflows, warrant deeper investigation.

Addressing these limitations constitutes an important direction for future work and will further strengthen the applicability of NIDS-β* in large-scale, real-world cybersecurity environments.

## 5 Conclusion

This work introduced NIDS-β*, an intelligent and explainable network intrusion detection framework inspired by transformer-based architecture. By combining contextual representation learning with traditional flow-level statistics, the proposed approach aims to enhance the detection of complex cyber threats while improving model interpretability. Experimental evaluations conducted on the UNSW-NB15 and CIC-IDS2018 datasets demonstrate strong detection performance, with NIDS-β* achieving accuracies of 97.8 and 98.6%, respectively, consistently outperforming representative machine ML, DL and LLM-based baseline approaches. The proposed multi-head fusion mechanism, together with attention-based visualization and SHAP analysis, provides post-hoc explanations for most detection decisions, supporting improved transparency and analyst interpretability. While these explainability components facilitate insight into model behavior, their effectiveness has been validated within the scope of the evaluated dataset and offline experimental configuration. The results suggest that NIDS-β* represents a promising step toward more interpretable and operationally efficient intrusion detection systems, though further validation is required to assess scalability and robustness in real-world environments. Future work will focus on extending NIDS-β* toward practical *Security Operations Center (SOC)* deployment, including integration with *Security Information and Event Management (SIEM)* and *Security Orchestration, Automation, and Response (SOAR)* platforms, as well as investigating online learning for concept drift adaptation, federated and multi-agent settings, multi-modal data fusion, and RL–based adaptive thresholding. Additional research will focus on multi-modal data integration to enhance contextual awareness by jointly leveraging flow-level features with system logs, packet payloads, endpoint telemetry, and external threat intelligence feeds. This enriched representation is expected to support more informed and context-aware detection decisions Another direction of this work, we will experimentally validate the Control and Adaptation Plane under realistic streaming and time-sliced deployment settings, benchmarking drift detection across covariate, class-prior, and concept drift in terms of detection delay and false-drift alarms. We will then evaluate analyst-in-the-loop active learning to quantify label efficiency, analyst workload, and time-to-recovery after drift events. Finally, we will assess continual tuning guardrails (e.g., replay/regularization and calibration-aware retraining) to prevent catastrophic forgetting while maintaining calibration stability and acceptable runtime overhead.

Scalability and performance will also be evaluated in high-throughput and large-scale network environments to assess deployment feasibility. These directions aim to further evaluate and enhance the adaptability of the framework under dynamic and large-scale network conditions.

## Data Availability

The dataset analyzed during the current study are publicly available in the UNSW-NB15 repository, maintained by the Australian Centre for Cyber Security (ACCS). The dataset can be accessed at: https://research.unsw.edu.au/projects/unsw-nb15-dataset. The CIC-IDS2018 dataset used in this work is publicly available from the Canadian Institute for Cybersecurity (CIC) at the University of New Brunswick. The dataset can be accessed and downloaded from the CIC official website: https://www.unb.ca/cic/datasets/ids-2018.html.
